# Waste tea residue adsorption coupled with electrocoagulation for improvement of copper and nickel ions removal from simulated wastewater

**DOI:** 10.1038/s41598-022-07475-y

**Published:** 2022-03-03

**Authors:** Nizeyimana Jean Claude, Lin Shanshan, Junaid Khan, Wu Yifeng, Han dongxu, Liu Xiangru

**Affiliations:** School of Environment Northeast, Normal University, Changchun, 130117 China

**Keywords:** Environmental sciences, Energy science and technology, Engineering

## Abstract

The present research involves removing copper and nickel ions from synthesized wastewater by using a simple, cheap, cost-effective, and sustainable activated green waste tea residue (AGWTR) adsorption coupled with electrocoagulation (ADS/EC) process in the presence of iron electrodes. By considering previous studies, their adsorbents used for treating their wastewaters firstly activate them by applying either chemicals or activating agents. However, our adsorbent was prepared without applying neither chemicals nor any activating agents. The operating parameters such as pH, hydraulic retention time, adsorbent dose, initial concentration, current density, and operating cost for both metals were optimized. In ADS/EC, the removal efficiency was obtained as 100% for copper and 99.99% for nickel ions. After the ADS/EC process, Fourier transform infrared (FT-IR) spectroscopy, Scanning Electron Microscopy (SEM) and Energy-dispersive X-ray spectroscopy (EDS) analysis were used to characterize the adsorbent green waste tea residue. The adsorption isotherm and kinetic model results showed that the Langmuir and the pseudo-second-order were well-fitted to the experimental adsorption data better than the Freundlich and pseudo-first-order models for both Cu^2+^ and Ni^2+^ with their maximum adsorption capacity of 15.6 and 15.9 mg g^−1^, respectively. The above results give an option to recycle the metal-based industrial effluents, tea industry-based wastes, enabling a waste-to-green technique for adsorbing and removing the heavy metals and other pollutants in water.

## Introduction

People have begun to realize how essential excellent health is to them, and it is no longer merely important to have a standard lifestyle for mankind. Because there is a direct relationship between environment, health, and population, it is important to have a stable environment if we want to live a healthy life. Every day, the world's population grows, and this influences the ecosystem. The amount of water used increases as the population rises, which directly affects the water bodies and aggravates the issue of water^1^. Increased demand leads to the development of new industries and resources; more demands generate more industries and more resources. As a result of increased industrialization, heavy metal poisoning in water sources and its severe health effects on human society are becoming a major problem^2^. Because heavy metals are resistant to degradation and bioaccumulation in living and nonliving organisms, people in many nations are exposed to them through contaminated water. Heavy metals such as copper and nickel, as an example, have a negative impact on environmental sources^3^.

copper and nickel can be found in the amount of industrial effluents. Nickel allergies are common due to coming into touch with nickel-containing objects, and the carcinogenic effects of Ni^2+^ are well recognized^4^. Pollution of Cu^2+^ is caused by manning activities, electroplating, smelting, and the utilization of copper-based agrichemicals and manufacturing of brass. Copper is the most toxic metal to animals, and long-term inhalation of Cu-containing sprays has been linked to an increased risk of lung cancer in those who have been exposed^5^. At low levels, copper can play a significant role in human and animal metabolism. Too much copper, on the other hand, can cause serious side effects and toxicological concerns like convulsions, vomiting, cramps, and even death^6^. Nickel is a common metal in the environment, although little is known about its practical applications for humans and animals. Municipal and industrial waste and the usage of liquid and solid fuels all contribute to nickel pollution. Nasal cancer, contact dermatitis, headaches, allergies, lung fibrosis, cardiovascular and kidney damage, and other toxicological concerns are all caused by it^4^. The World Health Organization (WHO) estimates the maximum permitted concentrations of Cu^2+^ and Ni^2+^ in drinking water to be less than 2 mg L^−1^ and 0.02 mg L^−1^, respectively^7^. So, the removal of both metals from polluted water is critical. Heavy metal removal from contaminated water is done in different techniques, including anaerobic biological treatment^8^, sonolysis^8^, and photocatalytic and oxidation destruction via ultraviolet/ozone treatment^9^, flocculation and coagulation^10^, adsorption^11^, biodegradation^12^ and electrocoagulation^13^, etc. However, these methods have several drawbacks, including low removal efficiency, high running costs, and the production of a lot of sludge, which is not environmentally friendly. Thus, to reduce potential pollution, high operating costs and low efficiency, the best answer to this problem is to combine two or more efficient techniques. In this study, the integration of adsorption (ADS) and electrocoagulation (EC) methods are suggested as an interesting alternative method to treat polluted water and wastewater.

Heavy metal ions can be eliminated from contaminated water through the adsorption process, which is proven effective. Due to its efficiency and low cost, ADS has been recommended to remove Cu and Ni metals from water and wastewater. It does not produce secondary sludge^14^. Adsorbents such as lignocellulosic biomasses^15^ fly ash^16^, powdered marble wastes^17^, activated carbon^18^, clays and biochars^19^ have all been used to remove contaminants, including metal ions in the adsorption method. The adsorbents discussed above have drawbacks, such as low efficiency, high cost, and limited availability. Our solution is to use activated green waste tea residue (AGWTR) as a long-term, low-cost, and more effective adsorbent. It is easily accessible on a huge scale throughout the world. No activating chemicals, coatings, or modifications were used to activate our material (AGWTR). In this work, we chose AGWTR to decrease the barriers associated with dangerous chemicals, high cost-effective and lengthier preparation processes to obtain the best removal efficiency of heavy metal treatment through the use of an environmentally benign and sustainable adsorbent. This is the key research topic, and it involves activation, which has a high adsorption capacity of^20^.

The electrocoagulation process needs simultaneous metal dissolution from the anode electrode and hydrogen gas and hydroxyl ions production at the cathode electrode. All contaminants that can be eliminated by electrocoagulation include total organic carbon, heavy metals^21^, antibiotics and medicines^22^, and organic pollutants such herbicides, phenols, and textile dyes^23^. A sacrificial metal anode and a cathode are the two electrodes in an electrochemical cell. The anodes in our study were iron (Fe) electrodes. The EC creates iron ions from a sacrificial anode, which hydrolyze in water and produce various coagulant species. Coagulation is the process of combining these coagulant species to generate bigger particles^24^. Coagulation is a process that uses coagulant chemicals to destabilize particles and allow them to bind to other particles. Iron salts were hydrolyzed in water to produce insoluble precipitates, which then adsorb on the surface of the particles, destabilizing their charge. Because the particles have identical electric charges, which are usually negative, they have repulsive interactions^25^, found that the hydrolyzed products had a positive electric charge. On the surface of the cathode, electrolytically created gases, primarily hydrogen, are produced. Gas bubbles are produced as a result of favorable side reactions, which aid in floating. These agglomerated pollutants form more agglomerates, which push higher and are destroyed in the next step. On the cathode, a final electrochemical reaction called reduction may occur^21^. The electrochemical dissolution of the iron anode is much more complex because there are two oxidation states of iron species: Fe^2+^ and Fe^3+^ (Eq. 1). According to solution pH and the dissolved oxygen, Fe^2+^ species can be potentially oxidized to the Fe^3+^ (Eq. 2) and finally hydrolyzed to form the hydroxide (Eq. 3)^26^.

1 $${\mathrm{Fe}}_{(\mathrm{s})}\to {\mathrm{Fe}}_{(\mathrm{aq})}^{2+}+{2\mathrm{e}}^{-},$$

2 $${4\mathrm{Fe}}_{(\mathrm{aq})}^{2+}+{\mathrm{O}}_{2}+{4\mathrm{H}}^{+}\to {4\mathrm{Fe}}_{(\mathrm{aq})}^{3+}+ {2\mathrm{H}}_{2}\mathrm{O},$$

3 $${\mathrm{Fe}}_{(\mathrm{aq})}^{3+}+ {3\mathrm{H}}_{2}\mathrm{O }\to {\mathrm{Fe}(\mathrm{OH})}_{3}+ {3\mathrm{H}}^{+}.$$

The main reaction at the cathode is hydrogen evolution (Eq. 4):

4 $${2\mathrm{H}}_{2}\mathrm{O }+ {2\mathrm{e}}^{-} \to {\mathrm{H}}_{2}+{2\mathrm{OH}}_{\left(\mathrm{aq}\right)}^{-}.$$

Based on the above discussions, we have chosen the combination of adsorption with electrocoagulation (ADS/EC) process as the most favorable methodology for wastewater treatment due to its simplest, less expensive, require low electricity and giving superior and optimistic results compared with adsorption or electrocoagulation method alone. It offers the following advantages: (i) simplicity of operation, (ii) rapid sedimentation, (iii) low sludge production, (iv) environmental compatibility and tea waste is an attractive low material that can produce low-cost activated carbon.

This study aims to remove Cu^2+^ and Ni^2+^ from synthesized wastewater by combining the activated green waste tea residue adsorption without applying any chemical reagents with electrocoagulation techniques in the presence of an iron electrode at the lowest possible cost. Our proposed technique and raw material, such as AWTR adsorbent and Fe–Fe electrode, are better environmentally friendly and require low cost. Moreover, this provides a potential solution to the problems related to waste tea disposal and low-cost activated carbon production. Activated carbon made from waste tea has been discussed in few types of research, like Lokendra Singh Mukesh Parmar, Hakan Elebi et al., and others have reported removal efficiencies of 89%, 82%, 75%, and 76% for copper and nickel using tea waste, which are lower than our removal efficiencies shown in 3.2^27–31^. However, no other published studies have considered the preparation of activated carbon from waste tea without modifying or using activated reagents for removing heavy metals from wastewater. Thus, the feasibility of preparing activated carbon from waste tea without applying any chemical reagents was investigated in the current study. The comparison of ADS and EC integration for both single and binary systems and the total operating cost was investigated in this paper. The effects of pH, treatment time, adsorbent dose, initial concentration and current density on removal efficiency were studied in this study. Adsorption isotherms (Langmuir and Freundlich) and the kinetic modeling (pseudo-first-order, pseudo-second-order) of Cu^2+^ and Ni^2+^ were discussed. This study, therefore, gives an option to recycle the metal-based industrial effluents, tea industry-based wastes, enabling a waste-to-green technique for adsorbing and removing the heavy metals and other pollutants in water.

## Experimental section

### Materials

Green waste tea residue (GWTR) was purchased from Pinduoduo Inc., China. The AGWTR was produced using the physical activation processes. This activated carbon was derived from crushed GWTR biomass and then sieved, resulting in a good adsorbent with a particle size of 0.45 μm (mesh size) and finally heated at 500 °C in 2 h. Synthesized wastewater was prepared from stock solutions of 1000 mg L^−1^ Cu^2+^ ion obtained from copper chloride dihydrate (CuCl_2_·2H_2_O) and Ni^2+^ ion obtained from nickel chloride hexahydrate (NiCl_2_·6H_2_O). The solution of 0.1 M sodium hydroxide (NaOH) and hydrochloric acid (HCl) was used for pH adjustment and cleaning some materials. Iron electrodes bought from Taobao Inc. and DC power supply (Maisheng MS-605D) also were used during the electrocoagulation process. During the experiment, double distilled and deionized water were utilized.

### Experimental setup

**Figure 1:**
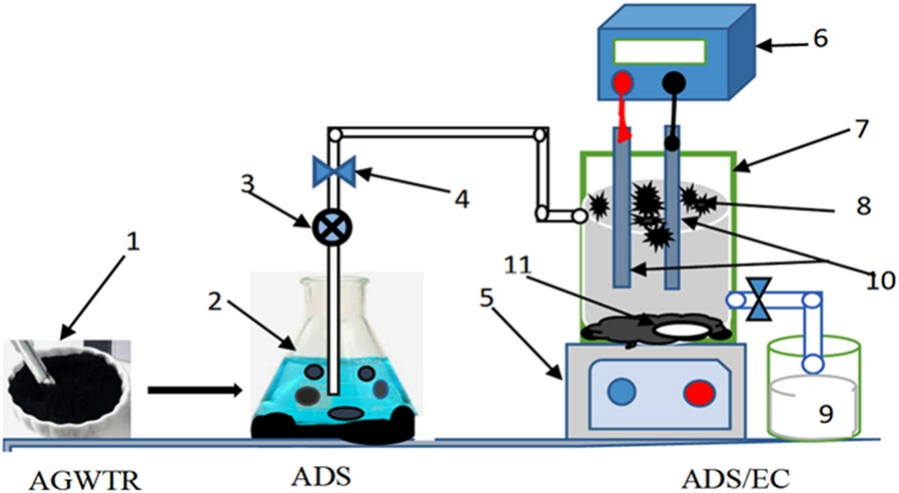
The apparatus layout used in the experiments: (1) AGWTR, (2) ADS Vessel; (3) pump; (4) control valve; (5) Stiller/Agitator; (6) direct current power source; (7) electro electric cell; (8) Flocs; (9) treated water in reception tank; (10) iron electrodes and (11) magnetic stiller.

The lab-scale batch experimental setup combined the ADS/EC studies schematically shown in Fig. 1. The EC cell was constructed from a thick glass container with 20 cm × 10 cm × 0.5 cm in length, width, and height, respectively. Copper and nickel solutions were agitated at 150 rpm (Agitator: Lichen DF-101Z) and the Temperature was kept constant at 25 ± 1 °C. The AGWTR was mixed with synthesized wastewater at various dosages (0.1–5 g L^−1^) in the combined system. The synthesized wastewater volume used in the experiment was 3 L. Iron (Fe–Fe) electrodes of 12 cm high, 7 cm wide and 0.2 cm in thickness were utilized for the sacrificial electrodes where Fe–Fe were used for both anode and cathode and also they were arranged in a monopolar configuration. The distance between electrodes was 1 cm. A peristaltic pump was also used in this experiment. The submerged surface area of electrodes was 84 cm^2^ and two plates were constructed in the electrochemical reactor. The ADS process in the presence of AGWTR-reaction occurred from the bottom of the reactor, while in the EC process, the electrodes-reaction happened at both the bottom and the top of the reactor. We were using a DC power source with a current of 0–5 A and a voltage of 0–30 V; a continuous, direct current was maintained for supplying current density.

### Preparation of the adsorbent

First, GWTR was thoroughly cleaned with distilled water to remove any adherent particles and then dried in a hot air oven at 100 °C for 2 h. Then the dried biomass was crushed using a crushing machine and sieved to get a good adsorbent with a fine particle size of 0.45 μm (mesh). Finally, sieved GWTR was placed inside the muffle furnace to heat it at 500 °C for 45 min in the N_2_ atmosphere to convert the green waste tea residue into activated carbon (biochar). After completion of the process, the activated green waste tea residue (AGWTR) was taken out and cooled for 5 h and stored in a plastic rubber for further experiment.

### Preparation of electrodes

Iron (Fe–Fe) plates of 12 cm × 7 cm × 0.2 cm dimension were utilized as electrodes in both anodes and cathode. The iron electrodes were washed in 0.1 M HCl to remove rust and other attached particles, then cleaned using a brush and distilled water before being dried in a dry oven at 70 °C for 10 min before they were used.

### Batch adsorption/electrocoagulation coupling process

The combining experiment was studied as follow: the amounts of activated green waste tea residue (GWTR) were tested and mixed with 3 L of synthesized solutions (1 L of copper, 1 L of nickel and 1 L of mixed of both metal solutions) in different Erlenmeyer flasks, then the ADS studies were conducted in 100 mL and each solution had a concentration of 20 mg L^−1^. The solution was stirred at 150 rpm and ambient Temperature was applied in this experiment. The sample was collected regularly from an electrocoagulation cell and then filtered using Whatman microfiber filter of 0.45 μm pore size before being analyzed by atomic absorption spectrophotometer (AAS Z-5000, Japan). The pH was maintained continuously using a multi-parameter instrument (SX725 pH/mV/DO meter). The characteristic of mineral composition, surface morphology and bonding patterns of a compound found in AGWTR after ADS and also compound observed in sludge after EC were evaluated by using the Fourier transform infrared (FT-IR-Magna 560) spectra, Scanning electronic microscope (SEM), and EDS analysis (JEM-2100F, 200 kV). To confirm the optimum level of high removal efficiency, the experiment was repeated at different levels. Effect of various parameters have been investigated during the lab experiment treatment time (10–180 min), pH (2–8), agitation speed (50–200 rpm), adsorbent dosage (0.1–5 g), initial concentration of Cu^2+^and Ni^2+^ (20–120 mg L^−1^), Temperature (20–40 °C), current density (0.11–2.5 mA cm^−2^) and total operating cost. The percentage of copper and nickel ions removal was calculated from the following (Eq. 5)^32,33^:

5 $$\%R=\frac{{C}_{0}-{C}_{e}}{{C}_{0}}\times 100.$$

The mass balance equation was used to calculate equilibrium concentration, q_e_ (mg g^−1^) of Cu^2+^ and Ni^2+^ was given by^34,35^:

6 $${q}_{e}=\frac{V}{m} \left( {C}_{0}-{C}_{e}\right),$$

where *C*_0_ and *C*_*e*_ are the initial and equilibrium concentration of Cu^2+^ and Ni^2+^ in mg L^−1^, m is the amount of AGWTR (g) and V (L) is the volume of solution.

### Adsorption isotherm modeling

Adsorption isotherm models in the equilibrium state have proven a relationship between the amount of copper and nickel solution on AGWTR. Here are Langmuir and Freundlich's models were discussed in this study. Langmuir isotherm is valid for monolayer adsorption with the surface of activated green waste tea residue as adsorbent^14^, while the heterogeneous surface with multilayer adsorption is suggested by Freundlich model^36^. The equation below can be used to express the Langmuir isotherm:

7 $${q}_{e}=\frac{{q}_{m{K}_{L}{C}_{e}}}{1+{K}_{L}{C}_{e}}.$$

By formulating the Langmuir equation to a linear form, the adsorption parameters of Langmuir were obtained.

8 $$\frac{1}{{q}_{e}}=\frac{1}{{q}_{m }{K}_{L}{C}_{e}}+\frac{1}{{q}_{m}},$$

where q_e_ is the amount of metal ions (Cu^2+^ and Ni^2+^) adsorbed per gram of the AGWTR at equilibrium (mg g^−1^), q_m_ the maximum monolayer coverage capacity (mg g^−1^), K_L_ is Langmuir isothermal constant (L mg^−1^) and C_e_ is the equilibrium concentration of adsorbate (mg L^−1^). q_m_ and K_L_ were determined from the slope and intercept of the Langmuir plot of 1/q_e_ versus 1/C_e_. The important features of the Langmuir isotherm model can be computed in terms of equilibrium parameter R_L_, which is a dimensionless constant denoted as equilibrium parameter or separation factor.

9 $${R}_{L}=\frac{1}{1+{K}_{L}{C}_{O}},$$

where C_o_ is the initial concentration of metal ion (mg L^−1^), K_L_: Langmuir isotherm constant). R_L_ value indicates the adsorption nature either unfavorable (R_L_ > 1), linear (R_L_ = 1), favorable (0 < R_L_ < 1) and irreversible (R_L_ = 0).

Freundlich isotherm model is commonly applied to describe the process of adsorption features of the heterogeneous surface. The linear equation of Freundlich isotherm is expressed below:

10 $$ln{q}_{e}=\frac{1}{n}ln{C}_{e}+ln{K}_{F},$$

where K_f_ is Freundlich isotherm constant (mg g^−1^), q_e_: the amount of adsorbed metal gram of the adsorbent at equilibrium (mg g^−1^), C_e_: the equilibrium concentration of adsorbate (mg L^−1^) and n: adsorption density.

### Adsorption kinetic modeling

To study kinetics that removed metal ions from a solution, the degree of adsorption was calculated as a function of time. In this study, the concentration of Cu^2+^ and Ni^2+^ at time t, q_t_ (mg g^−1^) was computed. The kinetic data were examined by using pseudo-first-order and pseudo-second-order models. The pseudo-first-order kinetic model was developed and expressed as follows:

11 $$\mathrm{ln}\left({q}_{e}-{q}_{t}\right)=ln{q}_{e}-{K}_{1}t,$$

where, q_t_: the amount of adsorbed metal ion per gram of adsorbent at any time (mg g^−1^), q_e_: the amount of metal ions adsorbed per gram of adsorbent at equilibrium (mg g^−1^), K_1_ = the adsorption rate constant (min^−1^) and t is a constant time (min). The adsorption rate constant K_1_was computed from the slope of the graph drawn $\mathrm{ln}({q}_{e}-{q}_{t})$ against t and the theoretical ${q}_{e}$ was computed from the breaking point on the graph.

While the pseudo-second-order kinetic model was developed and expressed as follows:

12 $$\frac{1}{{q}_{t}}= \frac{1}{{K}_{2}{q}_{e}^{2}}+\frac{t}{{q}_{t}},$$

where, q_t_: the amount of adsorbed metal ion per gram of adsorbent at any time (mg g^−1^), q_e_: the amount of metal ions adsorbed per gram of adsorbent at equilibrium (mg g^−1^), K_2_ = the adsorption rate constant (g mg^−1^ min^−1^), K_2_ q_e_^2^ is initial adsorption speed and t is a constant time (min). The adsorption rate constant K_2_ and theoretical q_e_ values are calculated respectively from the slope and breakpoint of the graph drawn t/q_t_ against t.

### Energy consumption and amount of dissolved electrodes

Energy consumption is a very important cost factor of the electrocoagulation treatment process. It is proportional to the electric current and applied voltage^37^. The following equation calculated the energy used for removing Cu^2+^ and Ni^2+^:

13 $$\mathrm{w}\left(\mathrm{kWh }{\mathrm{m}}^{-3}\right)=\frac{\mathrm{I }\times \mathrm{t }\times \mathrm{v}}{\mathrm{V}},$$

where W is energy consumption (kWh m^−3^), I is the electric current (A), v is applied voltage (volt), t is the reaction time (h) and V is the sample volume (m^3^). Additionally, Faraday's law describes the mass of iron electrodes dissolved in the solution^38^. It is shown as:

14 $${m}_{Fe}=\frac{I\times t\times {M}_{w}}{ZF},$$

where m_Fe_ is the mass of dissolved iron electrode (kg m^−3^), I am the current (A), t is the electrolysis time (s), M_w_ is the molecular mass of Fe (56 g g^−1^ mol), z is the number of electrons involved in the reaction Fe (2) and Faraday's constant (96,485.34 C mol^−1^).

### Treatment cost (OC)

#### Operating cost (OP)

To calculate the viability of the electrocoagulation process, we considered operational cost (OC) as one of the response variables and computed this variable to be applied as the optimization methodology's aim. The equation of determining the operating cost (OC) process was expressed as follows^39^.

15 $$OC\left[\frac{USD}{{m}^{3}}\right]=\frac{\left(I\times v\times t\right)\times {C}_{kWh}+{C}_{PH}+\left(\frac{M\times I\times t}{z\times F}\right)\times {C}_{Fe}+{C}_{s}}{V},$$

where I and v are the electrical current and voltage used during process time t, respectively, C_kWh_ is the cost of electricity for the industrial sector by a local provider, C_pH_ is the cost of the hydrochloric acid (HCl) or Sodium Hydroxide (NaOH) needed to set the pH to the required condition, M is the molar mass of iron, z is the valency of iron in an ion form, F is the Faraday constant, C_Fe_ is the cost per kilogram of iron, and C_s_ is the cost of the treatment for the produced sludge. Finally, V is the volume of the sample treated under such conditions.

#### Operating cost of activation carbon (OC_AC_)

OC_AC_ was also calculated for this step, as shown in the equation below (Eq. 16) to reduce the final effluent's toxicity at the lowest possible cost^39^.

16 $${OC}_{AC}\left[\frac{USD}{{m}^{3}}\right]=\frac{{M}_{AC}\times {C}_{AC}}{V},$$

where, M_AC_ is the mass of activated carbon used during the adsorption and C_AC_ is the cost per mass unit of AC.

#### Total operating cost

The total cost of all treatment processes was computed as the sum of operating cost (OC) and operating cost of activated carbon (OC_AC_)^39^.

17 $${{\varvec{O}}{\varvec{C}}}_{{\varvec{T}}{\varvec{o}}{\varvec{t}}{\varvec{a}}{\varvec{l}}}\left[\frac{USD}{{m}^{3}}\right]=OC+{OC}_{AC}.$$

## Result and discussion

### Characteristic of adsorbent

#### Scanning Electron Microscopy (SEM) analysis and EDS analysis

**Figure 2:**
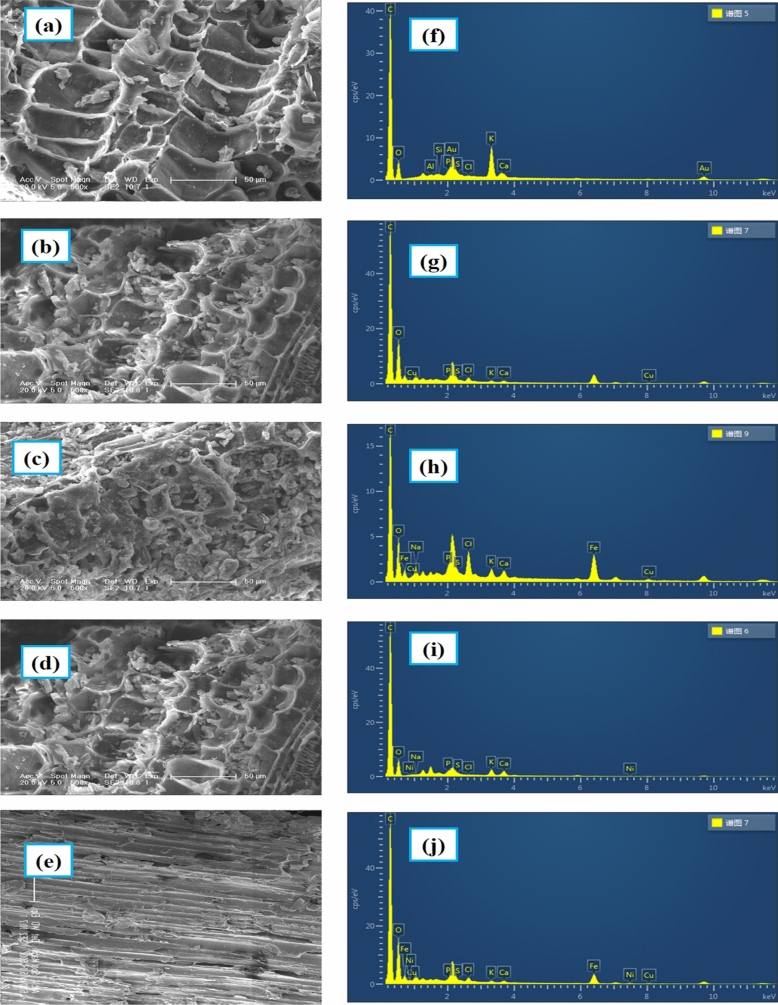
(**a**) SEM image of AGWTR before adsorption, (**b**) SEM image of AGWTR after adsorption of Cu^2+^, (**c**) SEM image of AGWTR after ADS/EC of Cu^2+^, (**d**) SEM image of AGWTR after adsorption of Ni^2+^, (**e**) SEM image of AGWTR after ADS/EC of Ni^2+^. (**f**) EDS spectra of AGWTR before adsorption, (**g**) EDS spectra of AGWTR after adsorption of Cu^2+^, (**h**) EDS spectra of AGWTR after ADS/EC of Cu^2+^, (**i**) EDS spectra of AGWTR after adsorption of Ni^2+^ and (**j**) EDS spectra of AGWTR after ADS/EC of Ni^2+^.

SEM was used to characterize the surface morphology of AGWTR. The SEM photographs of AGWTR before adsorption, after adsorption and after adsorption coupled with electrocoagulation of Cu^2+^ and Ni^2+^ removal are showed in Fig. 2a–e. The rough-stone-like with various larger holes have been observed in AGWTR before ADS Fig. 2a. There are rugged holes on external surfaces like crispy pits, which are visible. The rubbish surrounding those small holes causes the highest adsorption of both metals and they have been observed after adsorption of Cu^2+^ and Ni^2+^, respectively (Fig. 2b,d). At the same time, (Fig. 2c,e) show a plane highway-like smooth surface untimely. This means that the rough potholes that were observed in (Fig. 2b,d) for the AGWTR, was diminished and changed to smooth surface look like plane surface after copper and nickel adsorption coupled with electrocoagulation process; this means that Cu^2+^ and Ni^2+^ are absorbed maximally on AGWTR surface and also we confirmed that our material (AGWTR) showed the active site to adsorb Cu^2+^ and Ni^2+^ in maximum^33^.

As observed in Fig. 2f–j, the EDS spectrum showed that the AGWTR contained C, Fe, O_2_, Al, Si, Na, Cu, P, Cl, Ni, K and Ca. The percentage of elements observed in AGWTR before adsorption is 79.89%, 17.52% and 1.92% for carbon, oxygen and potassium, respectively and the remaining elements are Si, P, S, Cl, Al, and Ca have the percentage of 0.69% (Fig. 2f). While Fig. 2g,i showed the morphology of AGWTR after the adsorption of copper and nickel, respectively. Some changes appeared on the surface of AGWTR, where we found new peaks of copper and nickel. Furthermore, the lack of a sodium peak in the EDS spectrum revealed that copper and nickel ions swapped Na^+^. Then, the EDS analysis of AGWTR after adsorption coupled with electrocoagulation (Fig. 2h,j) mainly represented some new peak of Fe, which was not observed in the first adsorption process due to the dissolution of Fe-electrode used during electrocoagulation in both copper and nickel ions removal spectrum. The images observed from SEM in this research display similar results as those published by Nikolic and Yildiz^40,41^.

#### Fourier Transform Infrared (FT-IR) analysis

**Figure 3:**
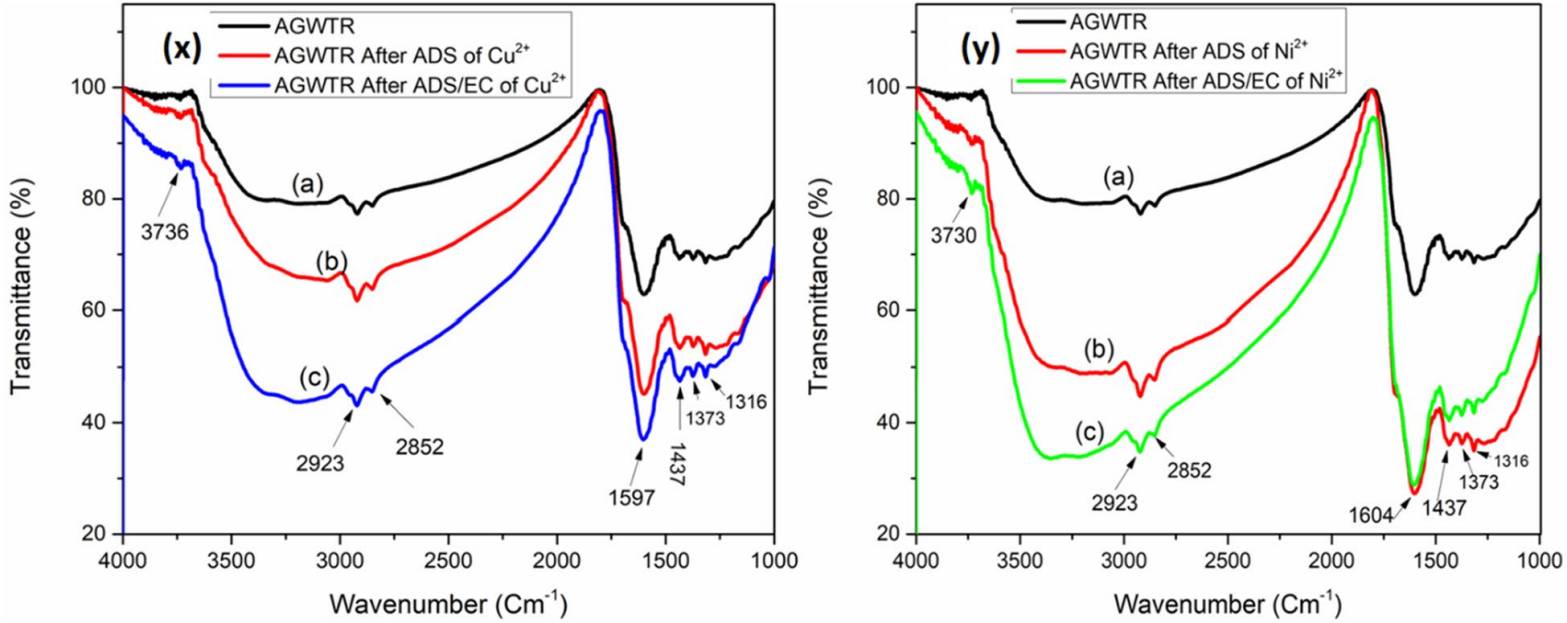
FT-IR spectroscopy characterization of activated green waste tea: (**X**) (a) AGWTR before adsorption, (b) AGWTR after adsorption of Cu^2+^ and (c) AGWTR after ADS/EC of Cu^2+^. (**Y**) (a) AGWTR before adsorption, (b) AGWTR after adsorption of Ni^2+^ and (c) AGWTR after ADS/EC of Ni^2+^.

To investigate the characteristics of functional groups of activated green waste tea as another adsorbent that is responsible for the Cu^2+^ and Ni^2+^ adsorption on its surface, Fourier Transform Infrared (FT-IR) spectrum analysis was performed. Figure 3x,y illustrate FTIR spectra of AGWTR before ADS (a), AGWTR after ADS (b) and AGWTR after coupling ADS/ EC (c) for both Cu^2+^ and Ni^2+^, respectively. In this research, the FT-IR spectrum of AGWTR was detected in the range of 1000–4000 cm^−1^. The FTIR spectrum of coupling ADS/EC for both copper and nickel absorption (c), there are seven distinct peaks, with a strong band of amine or hydroxyl (N–H or –OH) groups visible at wavenumber 3736 and 3730 cm^−1^. The band presented at 2923 cm^−1^ and 2852 cm^−1^ may indicate the –C–H stretching vibration from aliphatic compounds^42^. The aromatic ring vibration is observed at the sharp peak of 1604 cm^−1^^43^. The absorption at 1597 cm^−1^ indicated N–H bending in the adsorbent^44^. The C=N stretching in heterocyclic rings was also identified at wavenumber 1437 cm^−1^ while the peak appearing at 1373 cm^−1^ showed the deformation vibration of –C–H groups of alkanes^45^. The peak at 1316 cm^−1^ is due to the C–OH stretching vibration of alcohols and finally, the carboxylic acids were observed at peak 1156 cm^−1^ of the AGWT. By analyzing the relationship between these metal ions (Cu^2+^ and Ni^2+^) removal, we observed that AGWTR shows almost similar functional groups responsible for the Cu^2+^ and Ni^2+^ adsorption on its surface. Nevertheless, some little differences have been observed on both metal peaks of 1604 cm^−1^ and 1156 cm^−1^ found in Ni^2+^ and Cu^2+^ removal, due to interaction between Ni^2+^ and carbon aromatic structures and interaction between Cu^2+^ the carboxylic acid groups, respectively on the surface of AGWTR. As conclusion, Fig. 3x,y, have shown the presence of carboxylic acids, amines, and alcoholic aldehydes and amides groups in AGWTR has a great role in adsorbing of both copper and nickel ions from wastewater, and also the best removal of both metals ions have been observed on adsorption coupled with electrocoagulation band. These results showed also that AGWTR can remove Cu^2+^ and Ni^2+^ by both textural properties (microporosity and surface area) and their heterogeneous functional groups”.

### Effect of parameters

#### Effect of pH

**Figure 4:**
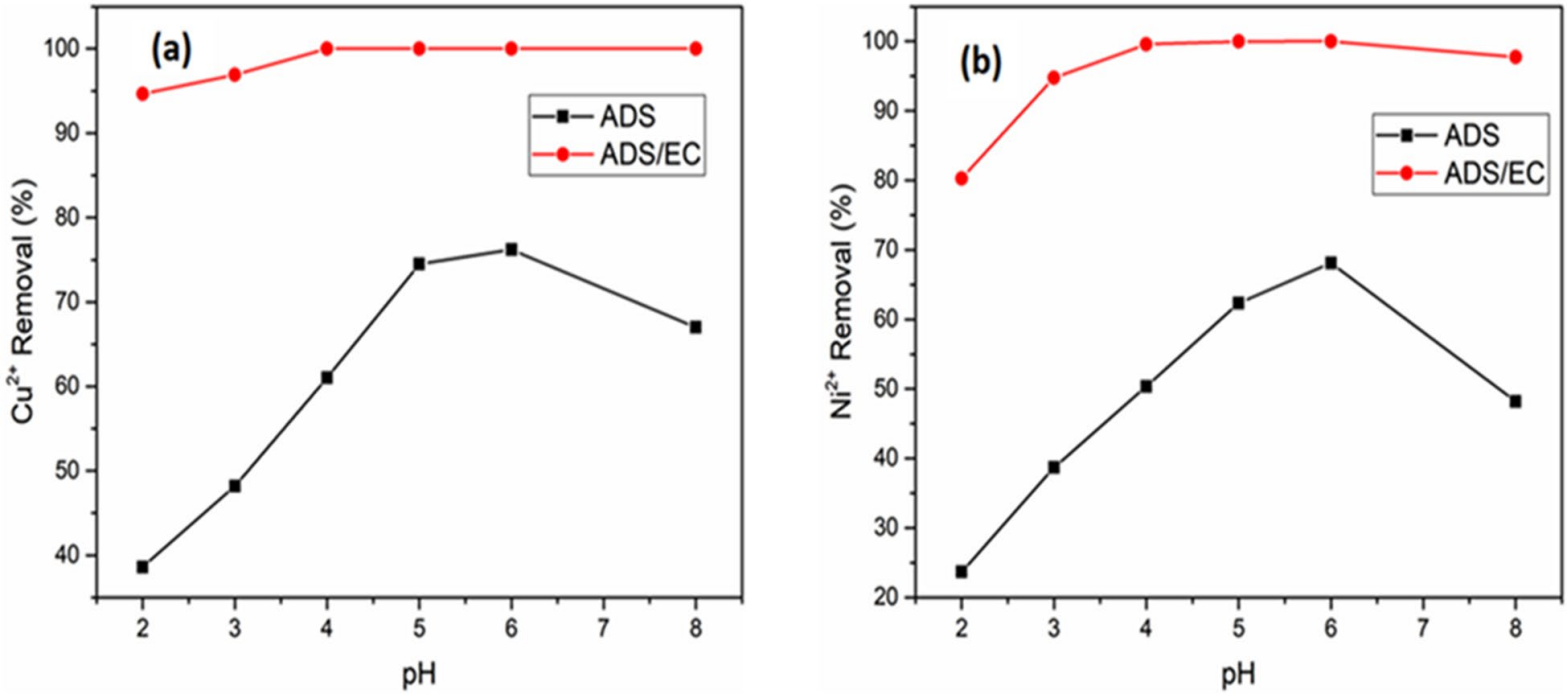
Effect of pH on (**a**) Cu^2+^ and (**b**) Ni^2+^ removal of the synthesized wastewater during the ADS and ADS/EC coupling processes (AGWTR dose = 1 g L^−1^, Initial conc. = 20 mg L^−1^ and Current density = 1.19 mA cm^−2^).

The effect of pH on the ADS and ADS/EC process of copper and nickel ions in the solution has been established and it has been considered the important parameter affecting the performance of ADS and ADS/EC process in water and wastewater treatment efficiencies. In this study, the pH value was adjusted in the range of 2–8. (Fig. 4a) shows that Cu^2+^ removal in the ADS and ADS/EC coupling process at different pH values, with an AGWTR dose of 1 g L^−1^ at the current density of 1.19 mA cm^−2^. The percentage of Cu^2+^ removal was a law at pH 2.0–4.0 during the ADS process because the solution was acidic, favoring the unseparated forms of functional groups^46^. The maximum removal efficiency of Cu^2+^ for the case of ADS was 76.2% at pH = 6.0.

The same results were reported by Ref.^33^. While the ADS/EC process, the highest removal efficiency was achieved at pH = 4.0, due to the presence of Fe(OH)_3_ in the solution highly depends upon the pH and concentration of Fe^3+^ in the solution (Eq. 3). Generally, in ADS/EC, the removal efficiency occurs at low pH due to the precipitation of Fe(OH)_3_ happened in the EC reactor. Similar results were observed by Ref.^21^. In this process of coupling ADS/EC, the observed removal efficiency was 100%, with a current density of 1.19 mA cm^−2^. Processes used in copper removal for studying the effect of pH, are similar to nickel removal. Figure 4b shows the equilibrium removal efficiency of Ni^2+^ during the ADS and ADS/EC processes. The removal efficiency for Ni^2+^ was 68.2% at pH = 6.0 during ADS, while when we applied ADS/EC, the removal efficiency was 99.98% at pH = 4.0. As we have seen in these results, the removal efficiency of Cu^2+^ is higher than Ni^2+^ for both cases (ADS and ADS/EC). Therefore, considering the removal efficiency of both metal ions, when we increase pH from 4.0, the amounts of OH are increased in solution. Some of the hydroxide ions oxidized at the anode. This reaction inhibits the production of the same amount of iron ions, consequently, the removal of Cu^2+^ and Ni^2+^ decreased. So, the original pH of the synthetized wastewater (i.e. 4.0) can be chosen because there are no chemicals required. Therefore, due to the production and the consumption of those hydroxide ions from reaction, the removal of Cu^2+^ and Ni^2+^ in solution increase during ADS combined EC when the initial pH is low^47^. From the above explanations, we can conclude that the initial pH after mixing the AGWTR with adsorbate increases the increases of Cu^2+^ and Ni^2+^ removal compared to both ADS and EC alone. The same results have reported in Ref.^48^.

#### Effect of electrolysis time

**Figure 5:**
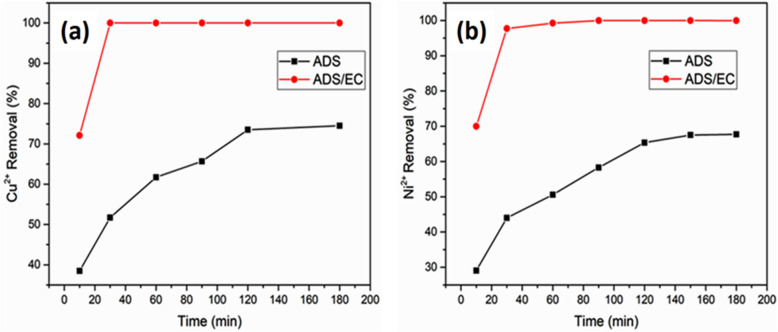
Effect of time on (**a**) Cu^2+^ and (**b**) Ni^2+^ removal synthesized wastewater during the ADS and ADS/EC coupling processes (activated green waste tea dose = 1 g L^−1^, Initial conc. = 20 mg L^−1^ and Current density = 1.19 mA cm^−2^).

Time is an important parameter that influences the water and wastewater treatment efficiency of ADS and ADS/EC coupling processes. As shown in Fig. 5, the effects of electrolysis time on copper and nickel removal were studied in the range of 10 min up to 180 min at an optimum current density of 1.19 mA cm^−2^. Both metals Cu^2+^ and Ni^2+^ during the adsorption process, the observation reveals that the Cu^2+^ and Ni^2+^ removal increases with an increased adsorption contact time due to the presence of natural material of AGWTR as shown in Fig. 5a,b. At 120 min of contact time, 1 g of AGWTR removed 73.51% of Cu^2+^ and 66.01% of Ni^2+^, respectively. This increase in both Cu^2+^and Ni^2+^ adsorption at the beginning of the ADS process was happened because of the high availability of active surface sites on the AGWTR surface. When these available sites were readily occupied, the following slow ADS is generally affected by diffusion into the interior pore spaces of AGWTR^49^.

In general, the generated coagulant from iron electrodes increases with reaction time for the ADS coupled with the EC process. The dissolution amount of iron was directly proportional to electrolysis time and therefore, the quantity of Fe^2+^ or Fe^3+^ ions and their flocs increased with the electrolysis time, resulting in higher removal efficiencies due to sweep coagulant and co-precipitation^50^. The removal efficiency of Cu^2+^ and Ni^2+^ increases with an increase of reaction time until it reaches a maximum of 30 min as the optimum time, as observed Fig. 5. This Fig. 5a,b showed that a total of 100% and 99.98% of copper and nickel ions were respectively removed in the synthesized wastewater at 30 min with a current density of 1.19 mA cm^−2^. The removal of both metal ions Cu^2+^ and Ni^2+^ with electrolysis time occurred through the production of Fe(OH)_3_ "sweep flocs" which have large surface areas that are favorable for faster adsorption of Cu^2+^ and Ni^2+^ and adsorption of soluble organic compounds from wastewater into flocs that holds many complexes of ferric polymeric hydroxide which are responsible for eliminating the pollutants^51^.

#### Effect of AGWTR dose

**Figure 6:**
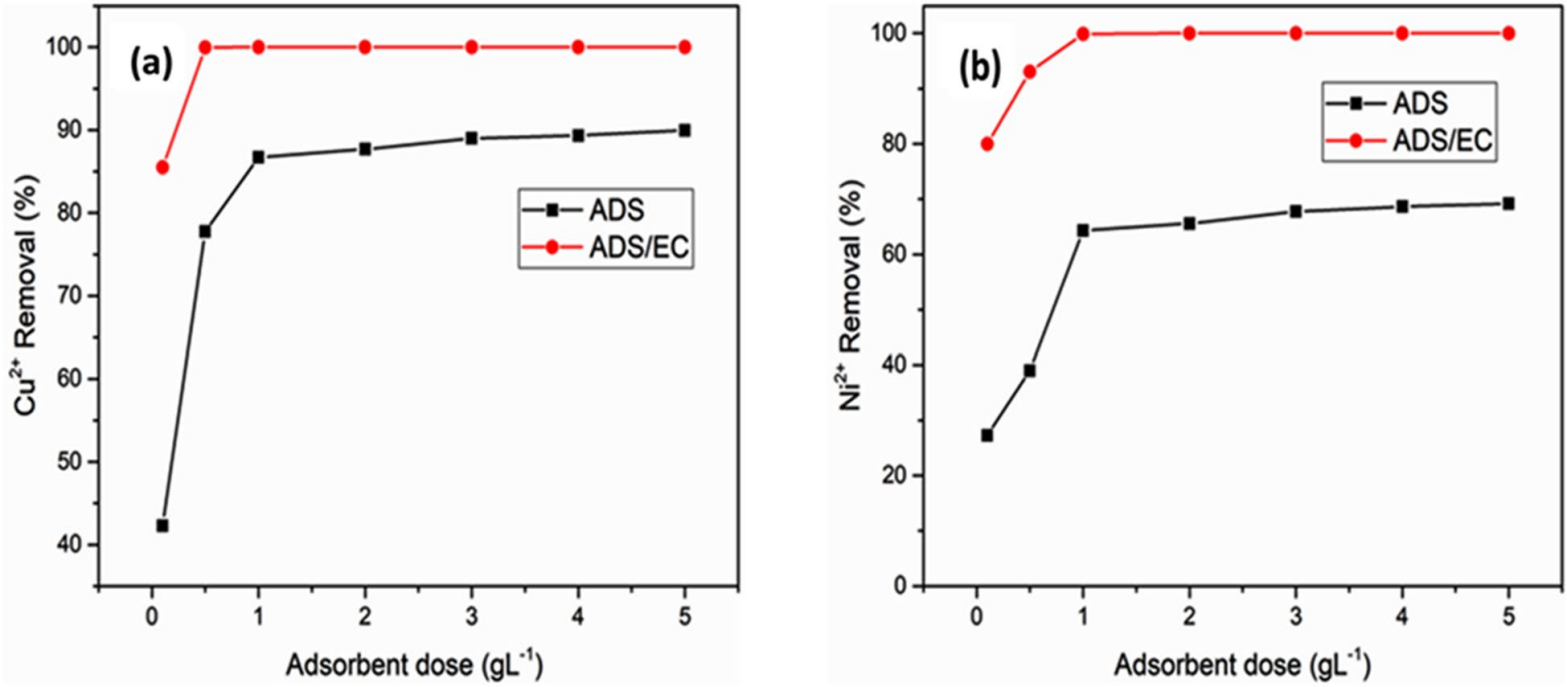
Effect of Adsorbent dose on (**a**) Cu^2+^ and (**b**) Ni^2+^ removal of the synthesized wastewater during the ADS and ADS/EC coupling processes (Initial conc. = 20 mg L^−1^ and Current density = 1.19 mA cm^−2^).

In this work, the effect of various adsorbent doses on the removal efficiency of copper and nickel for simple ADS and ADS/EC coupling processes at different AGWTR doses were presented in Fig. 6a,b and the adsorbent was ranged in between 0.1 and 5 g L^−1^ during 120 min. The ADS experiments show that the efficiency of both metal ions (Cu^2+^ and Ni^2+^) removal increases with the increase of the adsorbent dosage. In the ADS process, the removal efficiencies for both metals were 86.70% and 64.33% for copper and nickel, respectively, with 1 g L^−1^ of AGWTR for contacting time of 120 min. These results showed that with an increase of dosage, all copper and nickel ions in solutions may have interacted with the binding sites and then the highest Cu^2+^ and Ni^2+^ removal efficiencies are observed^52^. This may be happened due to the pretty number of ADS sites and more available surface area with the increase of adsorbent weight^53^. When AGWTR dosage is higher, the ADS process onto the AGWTR surface is very fast and Cu^2+^ and Ni^2+^ concentrations become lower in the solutions. While the ADS/EC coupling process compared to the simple ADS technique, the adsorbent was added in copper and nickel solution in the EC cell, resulting in a slow increase of Cu^2+^ and Ni^2+^ removal. When a dose of AGWTR increased up to 1 g L^−1^, the copper and nickel removal reached 100% and 99.99% for Cu^2+^and Ni^2+^, respectively, instead of 86.70% and 64.33% achieved during simple ADS. The ADS/EC occurred with both electrolysis time of 30 min and current density of 1.19 mA cm^−2^ for both metals removal. The metal removal percentages increase with an increase in the adsorbent doses and therefore, the adsorption sites on the AGWTR surface remain constant when the concentration of pollutants in the solution declines to the lowest value, as shown in Fig. 6a,b^54^. Here 1 g L^−1^ of AGWTR is enough to remove heavy metals in aqueous solutions at an instant level.

#### Effect of initial concentration

**Figure 7:**
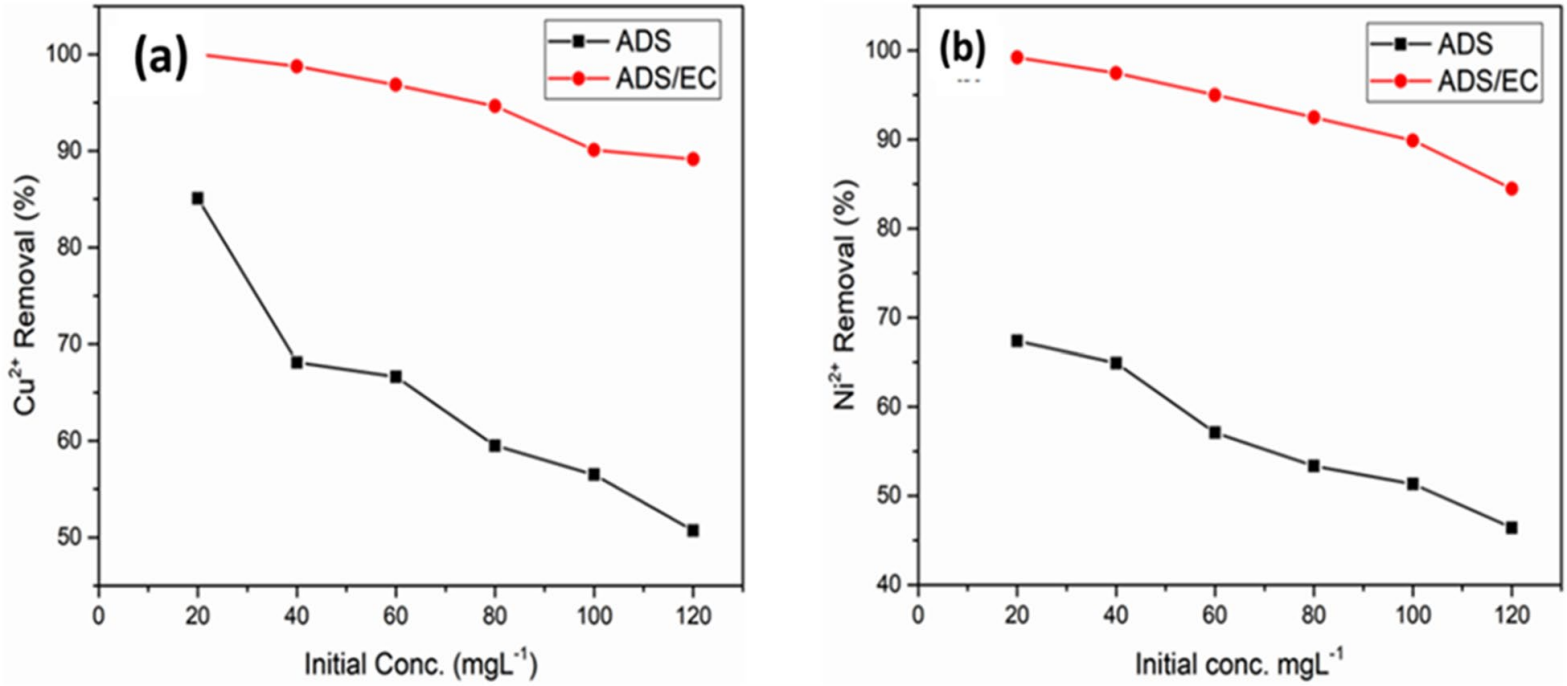
Effect of Initial Concentration on (**a**) Cu^2+^ and (**b**) Ni^2+^ removal of the synthesized wastewater during the ADS and ADS/EC coupling processes (AGWTR = 1 g L^−1^ and Current density = 1.19 mA cm^−2^).

The effects of the initial concentration on the Cu^2+^ and Ni^2+^ removal efficiencies at a constant AGWTR dosage of 1 g L^−1^ are observed in Fig. 7a,b. As planned, the Cu^2+^ and Ni^2+^ removal percentages were greater at lower initial Cu^2+^ and Ni^2+^ concentrations in the solutions for both processes (ADS and ADS/EC). For the case of ADS, we have investigated the maximum removal efficiency for both metal ions, by studying their different initial concentrations in their solutions. The experiments are conducted to the different Cu^2+^ and Ni^2+^ concentrations of 20, 40, 60, 80, 100 and 120 mg L^−1^. In this study, the removal percentage of copper and nickel ions was 85.08% and 67.37%, respectively, in the adsorption process. These results were shown that the removal efficiencies were decreased with increasing Cu^2+^ and Ni^2+^ ion concentrations, as observed in Fig. 7a,b. This has happened because Cu^2+^ and Ni^2+^ ions have quickly adhered to the ADS sites and these adsorption sites resulted from the adsorption efficiencies become higher^55^. Similar results have been obtained by Farihahusnah Hussin^37^. For the ADS/EC coupling process, Cu^2+^ and Ni^2+^ was slightly decreased when their initial concentrations were beyond 20 mg L^−1^ because of the slowest coagulation rate; this means that the removal efficiencies were lower at the higher initial concentration values. Here, the removal efficiencies of both Cu^2+^ and Ni^2+^ ions in this process decreased from 99.98% to 89.13% and 99.20% to 84.46%, respectively, as increases from 20 to 120 mg L^−1^in a time of 30 min as was shown in Fig. 7a,b. The reason is that the number of coagulants generated was nearly the same and cannot be affected by initial concentration values. This quantity of sludge was unable to eliminate all initial Cu^2+^ and Ni^2+^ concentration values^56^. The same results have been discussed by Zakaria^57^.

#### Effect of current density

Current density is one of the most important parameters which affect the effectiveness of the ADS/EC coupling process; it is used to determine the gas bubbles production and the coagulant dosage rate, growth and size of the flocs^58,59^. Current density strongly affects both transfers of mass at the electrodes and solution mixing. To study the performance of ADS/EC coupling process for Cu^*2*+^ and Ni^*2*+^ removal, with 1 g of AGWTR and 2 iron (1 for anode and other 1 for cathode) electrodes, experiments (four solutions for each metal ion) were evaluated out at the optimum parameters of electrocoagulation process: Electrolysis time = 30 min, [Cu^2+^] and [Ni^2+^] = 20 mg L^−1^, pH = 4.0, inter-distance of electrodes = 1 cm, sacrificial area electrodes = 84 cm^2^ and stirring speed = 150 rpm. At low current density, an insignificant amount of anode dissolves and Cu^2+^ and Ni^2+^ removal decrease^21^.

**Figure 8:**
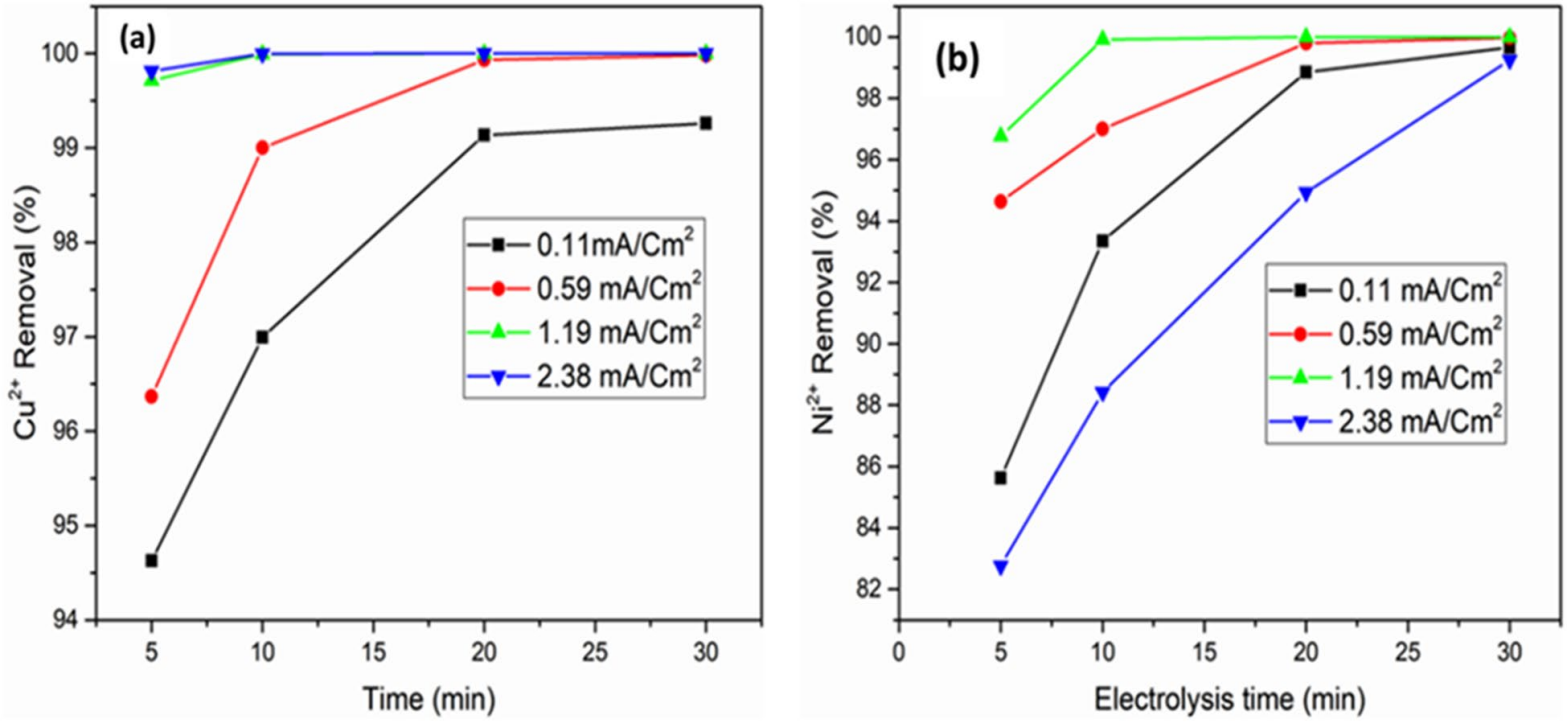
Effect of Current density on (**a**) Cu^2+^ and (**b**) Ni^2+^ removal of the synthesized wastewater during the ADS and ADS/EC coupling processes (Activated green tea waste dose = 1 g L^−1^, pH = 6, Current density = 1.19 mA cm^−2^, Initial conc. = 20 mg L^−1^ and contact time = 120 min).

In general, as it is known, the removal of metal ions from wastewaters increases with the increase of dosages of iron in chemical coagulation^60^. Both copper and nickel ions removal in ADS/EC is thus expected to be determined by the number of hydrous oxides produced in the solution. Faraday's law states that the mass of dissolved iron (Eq. 14) is directly proportional to current density (*j*)^48^. As a result, the creation of metal-hydrous ferric oxide complexes is typically used to define Cu^2+^ and Ni^2+^ elimination by ADS coupled with EC. Figure 8a,b displays that in 30 min, the removal efficiencies for both Cu^*2*+^ and Ni^*2*+^ are 100% and 99.99%, respectively and also these results showed that the removal efficiency of both metals are higher than those we found in the case of electrocoagulation alone. Thus coupling the ADS process to the EC on AGWTR is very effective and also, the target of the reduction of energy consumption was achieved. As we observed in the results, we found that with increasing current density, the removal efficiency for both metal ions increases due to higher dissolution of iron electrode material with higher formation rate of hydroxides resulting from co-precipitation. Also, production of more sludge is obtained from iron electrodes due to that higher rate of dissolution of the anode and those amounts of sludge enhance the removal of both Cu^2+^ and Ni^2+^ efficiency due to sweep at elevated current density. Additionally, the Fe–Fe electrode was found to be better compared to the other electrodes and the generation of more bubbles was observed at the higher current density of 1.19 mA cm^−2^ and increases both mixing and metal removal efficiency^26^. The other previous research has also reported that the metal ions removal efficiency increases with increasing current density^32,61^.

#### Isothermal study

**Figure 9:**
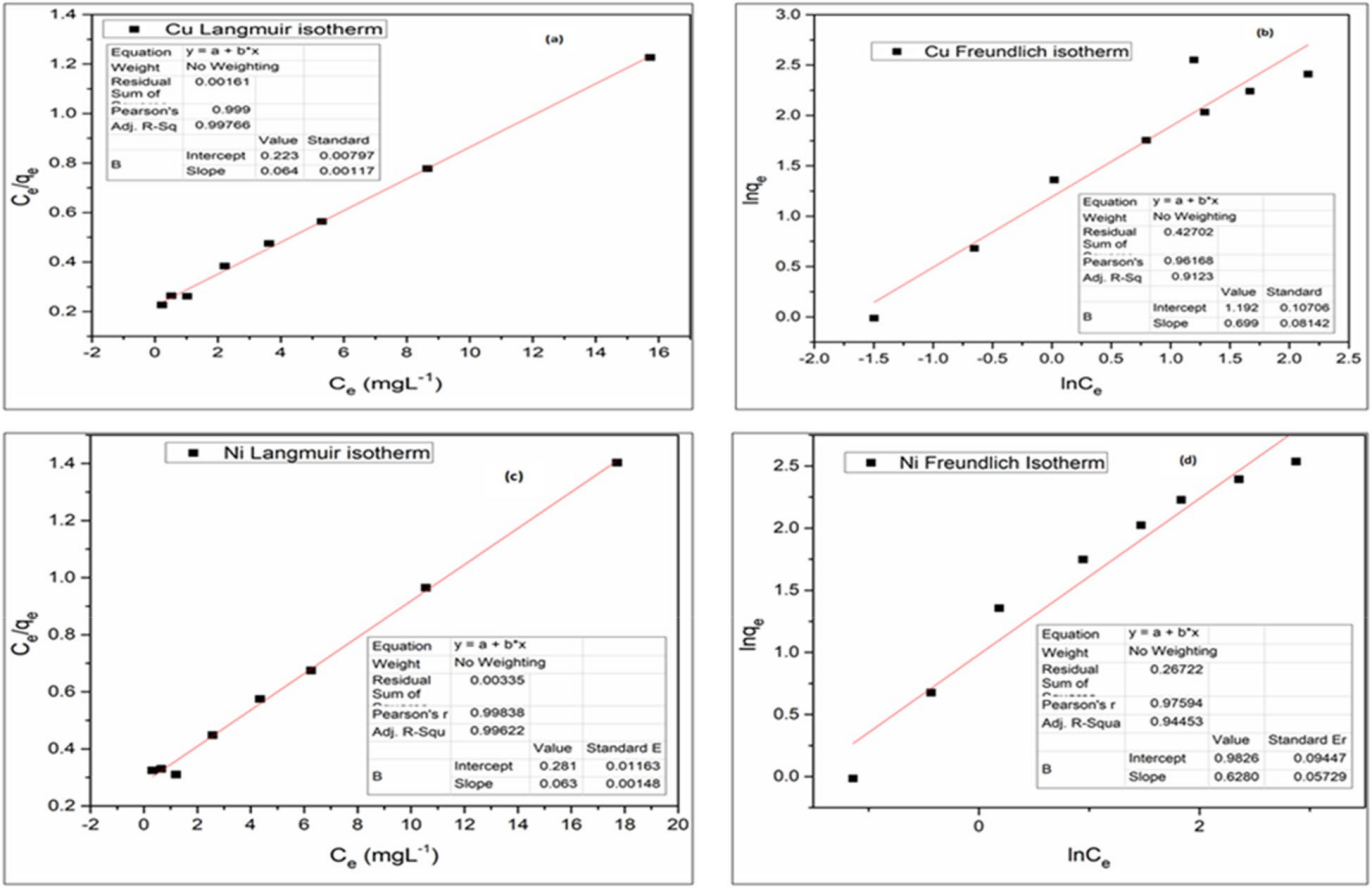
Adsorption/electrocoagulation isotherms: (**a**) Langmuir isotherm for Cu^2+^, (**b**) Freundlich isotherm for Cu^2+^, (**c**) Langmuir isotherm for Ni^2+^ and (**d**) Freundlich isotherm for Ni^2+^.

In combining ADS with EC, the adsorption isotherm for removing Cu^2+^ and Ni^2+^ from synthesized wastewater by AGWTR was investigated using Freundlich and Langmuir isotherm models. The correlation coefficients of both isotherm models are determined with their theoretical parameters and are observed in Table 1. The results obtained from experiments of both metal ion showed that Langmuir isotherm was the best-fitted model with higher regression coefficients of R^2^ = 0.997 and R^2^ = 0.912 compared to the Freundlich isotherms (R^2^ = 0.996 and R^2^ = 0.944) for copper and nickel ions, respectively (Fig. 9). The maximum regression coefficient indicated that copper and nickel ions are absorbed by AGWTR, forming a monolayer on its surface. For both copper and nickel ions, the maximum adsorption capacity of the adsorbent was estimated using the Langmuir isotherm, (${q}_{m}=$ 15.6 mg g^−1^) and ${q}_{m}=$ 15.9 mg g^−1^) at 25 °C, and were compared to several heavy metals removal found from the other researches, as observed in Table 2. These studies were carried out with a working volume of 100 mL of wastewater combined with 1 g of AGWTR, agitation speed of 150 rpm, and a working temperature of 30 °C, and an initial concentration of both Cu^2+^ and Ni^2+^ of 20 mg L^−1^ of the adsorbate. Here, Langmuir adsorption states that at particular sites of a homogenous surface of the AGWTR consisting of a fixed number of similar sorption sites, adsorption happens and the process of adsorption occurs as the saturation of these sorption sites^62^. The adsorption was limited to the monolayer layer coverage of both metal ions. Langmuir plot of Ce/q_e_ versus Ce was plotted in Langmuir isotherm and a straight line was observed. The Langmuir constant parameters q_m_ and K_L_ were computed using (Eq. 8) as mentioned previously are observed in Table 1. This also showed that the activated green tea waste residue and Fe-electrode are an effective, competitive adsorbent and low-cost for both Cu^2+^ and Ni^2+^ adsorption. We have seen also that the removal efficiencies and maximum adsorption capacities of both metals onto AGWTR and Fe-electrode in a real environment was also checked and the better results have observed in Table 3.

**Table 1:** Isotherm and Kinetic study parameters for Cu^2+^ and Ni^2+^ adsorption.

Cu^2+^ ions						Ni^2+^ ions					
Isotherm studies											
Langmuir isotherm			Freundlich isotherm			Langmuir isotherm			Freundlich isotherm		
q_m_ (mg g^−1^)	K_L_ (L mg^−1^)	R^2^	K_f_	n	R^2^	q_m_ (mg g^−1^)	K_L_ (L mg^−1^)	R^2^	K_f_	n	R^2^
15.6	0.4	0.997	0.15	0.8	0.912	15.9	0.4	0.996	0.2	1.02	0.944

Kinetic studies											
Pseudo-first order			Pseudo-second order			Pseudo-first order			Pseudo-second order		
${\mathrm{q}}_{\mathrm{e}}$ (mg g^−1^)	K_1_ (g g^−1^ min^−1^)	R^2^	${\mathrm{q}}_{\mathrm{e}}$ (mg g^−1^)	K_2_ (mg g^−1^ min^−1^)	R^2^	${\mathrm{q}}_{\mathrm{e}}$ (mg g^−1^)	K_1_ (g g^−1^ min^−1^)	R^2^	${\mathrm{q}}_{\mathrm{e}}$ (mg g^−1^)	K_2_ (g g^−1^ min^−1^)	R^2^
1.0392	1 × 10^–4^	0.945	0.0542	7.613	0.991	0.9770	3.06 × 10^–4^	0.926	0.0247	9.304	0.946

**Table 2:** Comparison of maximum adsorption capacities for heavy metals on some adsorbents*.*

Biomass/waste name	Activated agent	Created as adsorbent	Adsorbate	Max.adsorption capacity (mg g^−1^)	References
Green waste tea residue	No any activating agent	Without activation	Cu^2+^ and Ni^2+^	15.6 and 15.9	Present work
Sewage sludge	ZnCl_2_	Activated	Cu, Zn, and Al	15.58, 24.09 and 27.70	^68^
Bamboo SM1 SM2	No any activating agent	Activated	Cu(II), Cd(II) and Pb(II)	0.4, 0.17 and 0.40 0.4, 0.25 and 0.39	^69^
Pistachio shell	Fe_3_O_4_ NPs@AC@SO_3_H	Activated	Pb(II), As(III) and Cd(III)	147.05, 151.51 and 119.04	^70^
Sugarcane	ZnCl_2_	activated	Cu^2+^, Ni^2+^ and Pb^2+^	2.99, 13.24 and 19.3	^71^
Coconut shell	H_3_PO_4_	Activated	Cd(II)	33.71	^72^
Vegetable wastes	H_2_SO_4_, H_3_PO_4_ and NH_4_NO_3_	Activated	Cd^2+^, Ni^2+^ and Zn^2+^	4.97, 3.29 and 3.07	^73^
Sand	α-Fe_2_O_3_	Coated	Cu(II)	3.93	^35^
Sugarcane Bagasse	H_3_PO_4_	Activated	Hg	107.7	^74^

**Table 3:** Results of ADS/EC of Conc. Cu^2+^ and Ni^2+^ by AGWTR from different water samples.

Sr. no.	Water sample analyzed	Conc. Cu^2+^ and Ni^2+^ before ADS (mg L^−1^)		Conc. Cu^2+^ and Ni^2+^ after ADS (mg L^−1^)		Conc. Cu^2+^ and Ni^2+^ after ADS/EC (mg L^−1^)		Maximum adsorption capacity (qe) (mg g^−1^)		% Removal for ADS/EC process	
		Copper(II)	Nickel(II)	Copper(II)	Nickel(II)	Copper(II)	Nickel(II)	Copper(II)	Nickel(II)	Copper(II)	Nickel(II)
1	Changchun cars industry wastewater	107.1	84.5	41.7	33.6	2.03	2.4	10.507	8.247	98.104	97.7591
2	Xiao Hetai wastewater treatment plant	82.6	72.1	32.4	30.2	1.44	1.7	8.116	7.04	98.256	97.94189
3	Yinma River	61.3	40.03	23.8	21.4	0.17	1.03	6.113	3.9	99.722	98.31974

The Langmuir isotherm type was discussed and we found that the adsorption is favorable for adsorption of both Cu^2+^ and Ni^2+^ due to R_L_ (the dimensionless constant separation factor) (Eq. 9), which ranges from 0 < R_L_ < 1 and the values of our R_L_ were found to be 0.04 for both metals removal. Similar results were discussed: tartrazine adsorption onto Moringa oleifera seed^63^, dye adsorption onto tea waste^64^ and Methylene Blue Dye from industrial wastewater using prepared Activated Carbon^18^.

### Kinetic studies

**Figure 10:**
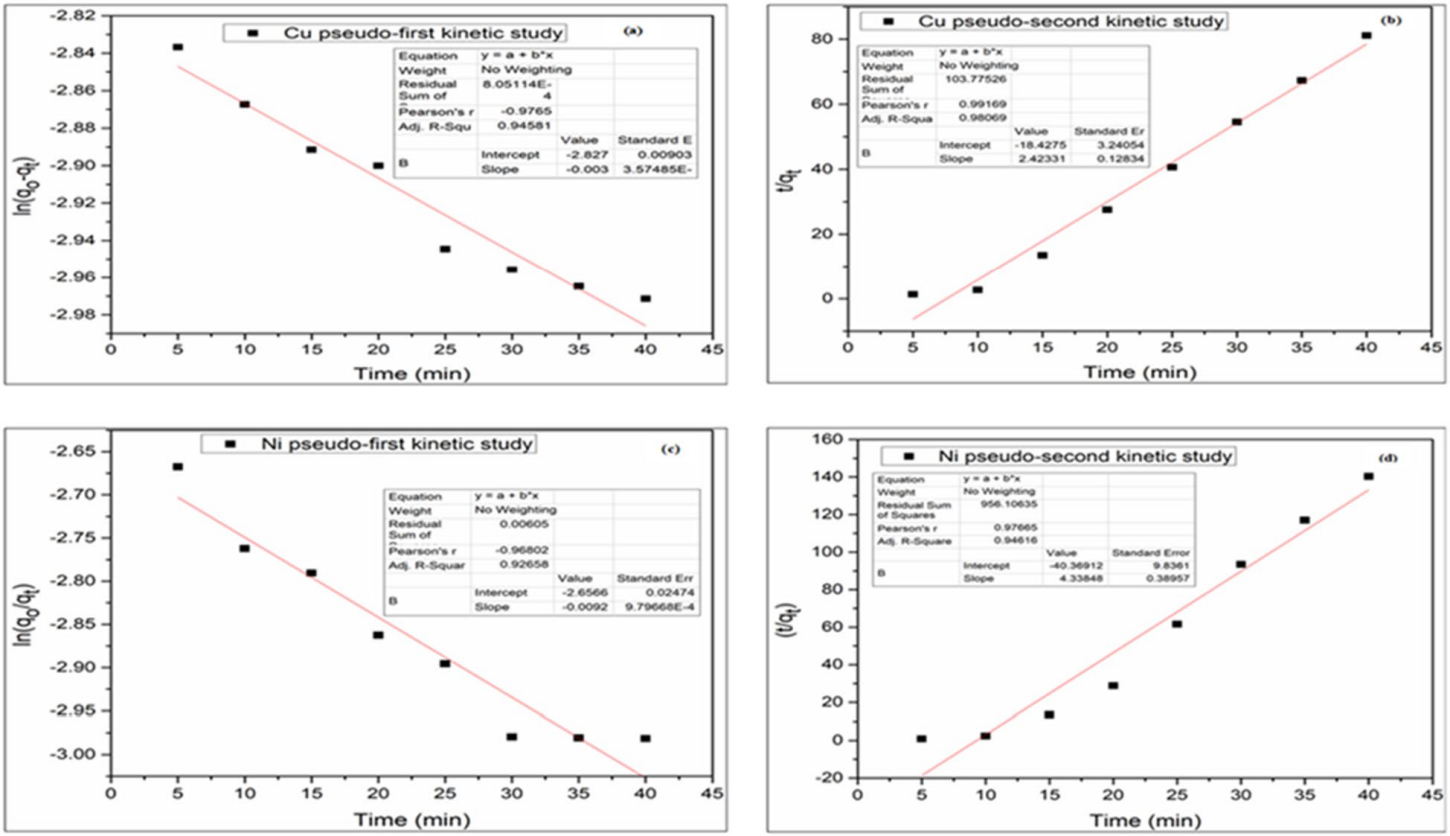
Adsorption/electrocoagulation Kinetic study (**a**) pseudo-first kinetic for Cu^2+^, (**b**) pseudo-second kinetic for Cu^2+^, (**c**) pseudo-first kinetic for Ni^2+^ and (**d**) pseudo-second kinetic for Ni^2+^.

To investigate the kinetics models for removing Cu^2+^ and Ni^2+^ by conventional ADS and ADS combining with EC process, the linear pseudo-first-order and pseudo-second-order kinetic models were analyzed to fit the experimental kinetic data. For pseudo-first-order, K_1_ was developed from the plotted slope of ln ($${q}_{e }-{q}_{t })$$ versus t (Eq. 11) while the pseudo-second-order kinetic model was plotted by taking $$\frac{t}{{q}_{t}}$$ Versus t (Eq. 12). They were tested according to the mentioned models and the correlation coefficients with the rate constants in both models are shown in Table 1. A linear relationship was calculated, K_2_ provided from intercept and qe (obtained from the slope) values were determined. The values of coefficient R^2^ derived from the plots of both pseudo-first-order and pseudo-second-order kinetic models were computed and the results were showed that the coupling ADS/EC process is led by pseudo-second-order of a kinetic model for both metal ions removal. For copper ions and Nickel ions removal, in pseudo-first-order kinetic model, w_e_ (1.055 mg g^−1^), K_1_ (0.006 h^−1^), R^2^ = 0.945 and q_e_ (0.977 mg g^−1^), K_1_ (0.0184 h^−1^), R^2^ = 0.926, respectively. For pseudo-second-order kinetic model, q_e_ (0.23 mg g^−1^), K_2_ (7.61 h^−1^), R^2^ = 0.980 for Cu^2+^ and q_e_ (0.15 mg g^−1^), K_2_ (9.3 h^−1^), R^2^ = 0.946 for Ni^2+^ could be observed from calculation. These facts showed that the pseudo-second equation model is the best fit to the experimental data with high R^2^ and also sorption of copper and nickel follow the pseudo-second-order kinetic model as shown in (Fig. 10), which meant that the adsorption rate was mainly regulated by chemisorption.

### Energy consumption and amount of dissolved electrodes

The energy consumption cost computations are necessary to calculate the feasibility of the ADS/EC application, not only to investigate the greatest interest of the metal removal efficiencies but also the consumption of power consumed for this technology application, as mentioned previously in Eq. (13), and (Eq. 14). Here, the energy consumed and the amount of dissolved iron electrodes have been calculated and the results are shown below: A current density of 1.19 mA cm^−2^, A = 84 m^2^ of electrode and a constant voltage of 8 V were used the optimum current density 1.19 mA cm^−2^. The reaction time was chosen to be 30 min and the volume was 100 mL = 0.0001 m^3^. Then, the energy consumption was calculated according to Hussin, where $\mathrm{w}\left(\mathrm{kWh }{\mathrm{m}}^{-3}\right)=\frac{\mathrm{I }\times \mathrm{t }\times \mathrm{v}}{\mathrm{V}}$ = $\frac{0.1\times 0.0001 \times 30 \times 8}{60\times 0.0001}$  = 0.4 kWh m^−3^ while m_Fe_ is the mass of dissolved iron electrode (kg m^−3^), M_w_ is the molecular mass of Fe (56 g g^−1^ mol), z is the number of electrons involved in the reaction Fe = 2 and Faraday’s constant (96,485.34 C mol^−1^), ${m}_{Fe}=\frac{0.1\times 30\times 56}{60\times 2\times 96485.34}$ = 1.4509 $\times$ 10^–5^ kg m^−3^. Therefore, W (kWh m^−3^) = 0.4 kWh m^−3^ and ${m}_{Fe}=$ 1.4509 $\times$ 10^–5^ kg m^−3^, respectively. Thus, these results are better than other results reported by other researchers, such as Refs.^54,65^.

### Competitive adsorption and electrocoagulation in binary metal systems

**Figure 11:**
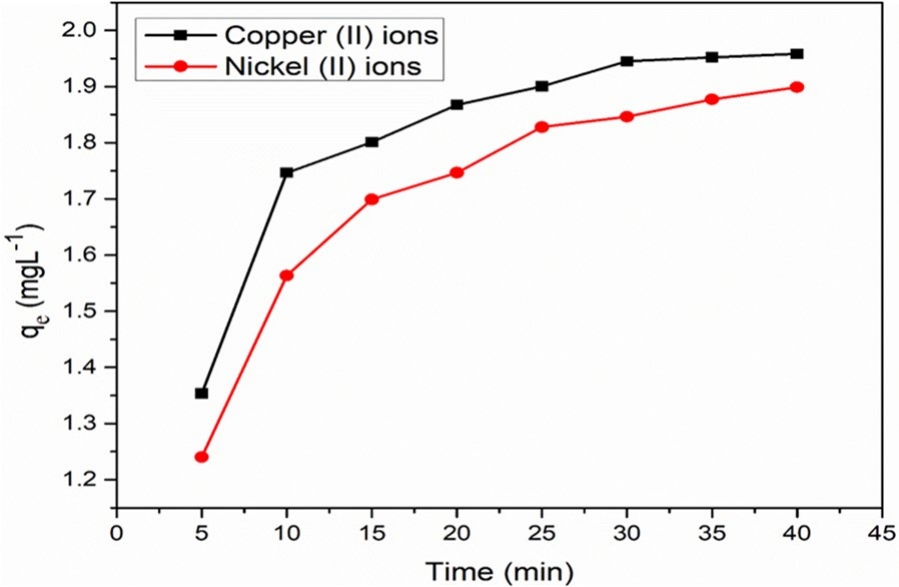
Combination of Cu^2+^ and Ni^2+^ ions in solution with 20 mg L^−1^ at T = 30 °C.

In this research, the competitive ADS/EC of copper and nickel ions in their binary solutions were studied similarly as described above. These experiments were investigated at a temperature of 30 °C at initial pH of 6.0. The main objective of this experiment was to investigate the effect of both Cu^2+^ and Ni^2+^ coexistence on the total capacity of adsorption in the presence of AGWTR combined with Fe-electrode. The result was observed in (Fig. 11). As observed in that figure, the values of the adsorbed amount of Cu^2+^ and Ni^2+^ are found and are described by referring to the following conditions (the initial pH of the solutions was kept at 6.0, 1 g of AGWTR per 20 mL of solution at 30 °C and reaction time of 30 min for both metals) which were ranging from 1.35 to 1.89 mg L^−1^and 1.22 to 1.83 mg L^−1^ for copper and nickel ions, respectively that were not greater than those for single-component solutions (1.44 to 2.0 mg L^−1^ and 1.39 to 1.99 mg L^−1^ for Cu^2+^ and Ni^2+^, respectively). Single metal ion present impeded through the uptake of another metal in the system, and both metals uptake were little lower than that in a single system. This showed that functional group of AGWTR surface have a relatively strongest affinity for copper ions than nickel ions.

### Reusability study

**Figure 12:**
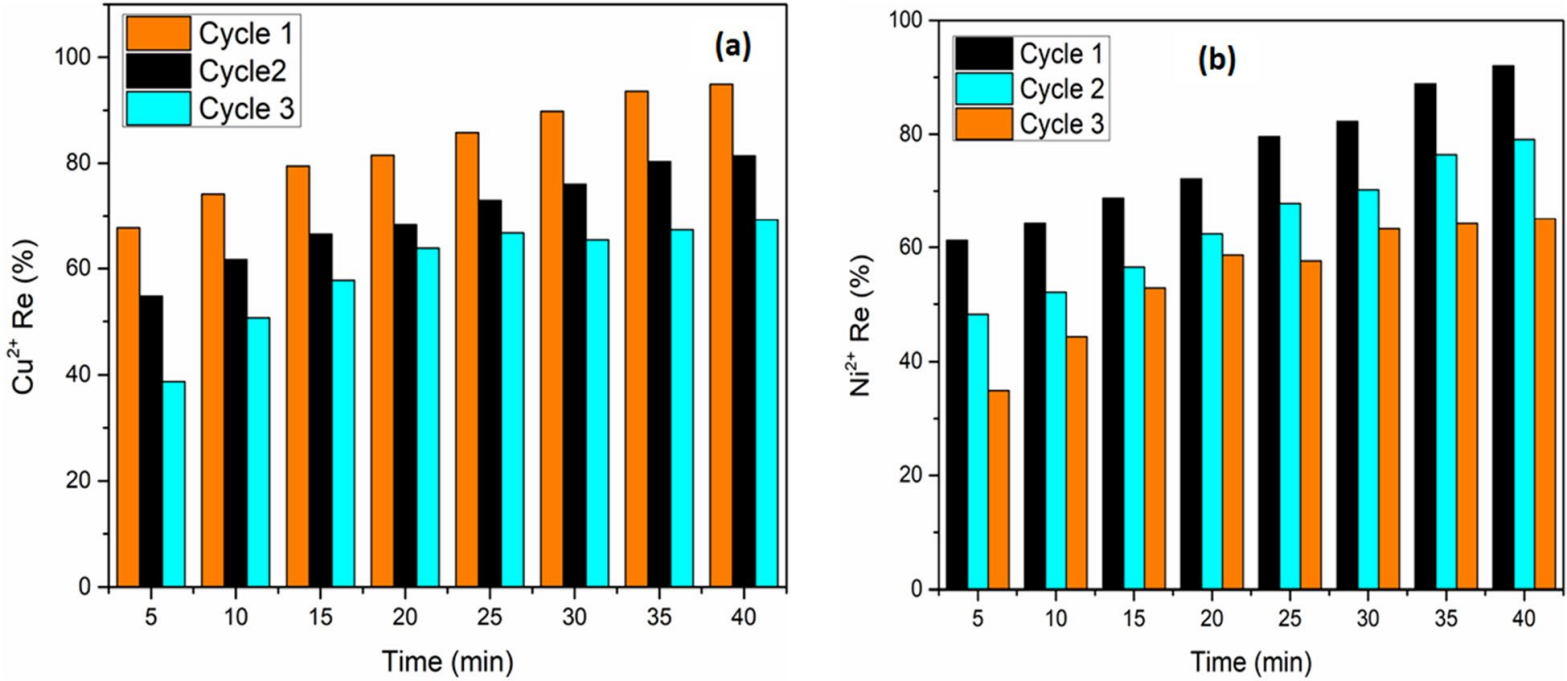
Maximum removal efficiency with reuse of the generated AGWTR for (**a**) Cu^2+^ and (**b**) Ni^2+^ removal using ADS/EC process. (AGWTR = 1 g; Cu^2+^ and Ni^2+^  = 20 mg L^−1^ at T = 30 °C).

The reusability of the adsorbent is a very important that aspect of the water treatment process. In this study of reusing material, especially AGWTR and Fe-electrode, we conducted ADS/EC processes using Cu^2+^ and Ni^2+^ as pollutants. ADS coupled with EC processes are similar to that we have discussed in experimental part 2.5. After ADS/EC, the sludge was separated by filtration procedure using Whatman microfiber filter of 0.45 μm pore size. To regenerate our materials, water and Hydrochloric acid of 0.1 M were used to wash Cu^2+^ and Ni^2+^ that AGWTR adsorbed at the previous stage and no solvent has been used here. As observed in Fig. 12a,b, the removal efficiency of Cu^2+^ and Ni^2+^ in three cycles are 94.87%, 81.39% and 69.23% and also 91.99%, 79.09% and 65.1% respectively. It is perfect, stable, easy and it can be applied repeated ADS coupled with EC of heavy metals. A cycle of this study were repeated three times as shown above for deciding the reusability potential of AGWTR and Fe as adsorbent and electrode, respectively.

### Treatment cost

The operational cost computations are necessary to calculate the feasibility of the ADS/EC application to investigate the greatest interest in the metal removal efficiencies and the most economical effect of this technology application. As we have discussed above, the total operation cost (${OC}_{Total}$) is the summation of different costs linked to the adsorption coupled with electrocoagulation process operation as it is showed by (Eq. 17). ${OC}_{Total}$ = (Energy consumption × Electricity cost) + (Electrodes consumption × Anode price) + chemical added × Chemicals price (the cost of the hydrochloric acid (HCl) or Sodium Hydroxide (NaOH) needed for pH adjustment) + cost of the treatment for the produced sludge or (${OC}_{Total}$) = The total cost of all treatment processes was computed as the sum of the cost of operating cost (OC) found in (Eq. 15) and operating cost of activated carbon (OC_AC_) for (Eq. 16)^39,66^. Here, C_kWh_ = 0.104 USD kWh^−1^, C_Fe_ = 0.0145 USD kg^−1^, C_s_ = 0.083 USD ton^−1^ or m^3^^67^, V = 0.0001 m^3^, Z = 2, Faraday’s constant (F) = 96,485.34 C mol^−1^, the molecular mass of Fe (M_w_) = 56 g g^−1^ mol, I = 0.1A, v = 8.0 V, t = 30 min, C_pH_ ≈ 0, M_AC_ = 1 g and C_AC_ = 0.00334 USD kg^−1^. Therefore, according to the Eqs. (15) and (16), the cost of operating cost (OC) has been calculated and it has fund to be 0.1246 USD m^−3^, including electricity price and transportation distance (max. 40 km), while the operating cost of activated carbon (OC_AC_) was 0.0334 USD m^−3^, respectively. Finally, the Eq. (17) was used to calculate the total cost of the adsorption combined with the electrocoagulation process, it was approximately 0.128 USD m^−3^, which is less than other costs found from other current studies^39,66^. Noticeably, the total operation cost (${OC}_{Total}$) depends on the prices of market of consumables and costs of sludge management and also the amount of water treatment technologies. Therefore, treatment of wastewater by this study (ADS/EC) respects Chinese standard costs.

## Conclusions

This work studied the removal of Cu^2+^ and Ni^2+^ from synthesized wastewater via ADS and ADS coupled with EC onto activated green waste tea residue. It was observed that our AGWTR is successfully proved as a cheap, cost-effective and sustainable adsorbent for heavy metal removal in synthetic wastewater. The operating parameters for both metal ions were: pH = 6.0, t = 120 min, AGWTR dose = 1 g and initial conc. = 20 mg L^−1^ for single ADS. While in ADS/EC process was optimized at pH = 4.0, t = 30 min, AGWTR dose = 1 g, initial conc. = 20 mg L^−1^ and *j* = 1.19 mA cm^−2^. Their removal efficiencies for Cu^2+^and Ni^2+^ were 73.51% and 66.01% in single ADS, while in ADS/EC process, 100% and 99.9% for Cu^2+^and Ni^2+^, respectively. Our AGWTR was prepared without applying neither chemicals nor any activating agents. The adsorption isotherm showed that Langmuir isotherm was the best-fitted model compared to the Freundlich isotherm model. The maximum adsorption capacity of AGWTR is obtained to be 15.6 and 15.9 mg g^−1^ for Cu^2+^ and Ni^2+^, respectively. The kinetic study of both metals followed a pseudo-second-order kinetic model. SEM shows folded cracks and various large holes on the external surfaces of the activated green tea residue, which look like the structure observed on GO generated from the graphite. Sorption on a single system was found to be more effective in producing desired or intended results than the one on the binary system and our results showed that the treatment cost is 0.128 USD m^−3^. Finally, based on the results found in this work, It is well understood that the use of the AGWTR adsorption coupled with EC technique is the cheapest compared with single ADS and EC technique for heavy metal removal due to remarkable low adsorption dose, energy consumption and it can be applied on a large scale for municipal and industrial wastewater treatment.

## References

[1] Jayasinghe HDRP, Riswan M, Ishaq P (2021). Water scarcity in Aligambai Village, Alayadivembu Divisional Secretariat, Sri Lanka. Shanlax Int. J. Arts Sci. Humanit..

[2] Si W (2015). Health risks of metals in contaminated farmland soils and spring wheat irrigated with Yellow River water in Baotou, China. Bull. Environ. Contam. Toxicol..

[3] Ali IH, Ateeg AA (2015). Study of soil pollutants in omdurman industrial area, Sudan, using X-ray fluorescence technique. Int. J. Environ. Res..

[4] Genchi G, Carocci A, Lauria G, Sinicropi MS, Catalano A (2020). Nickel: Human health and environmental toxicology. Int. J. Environ. Res. Public Health.

[5] Santos D (2020). Toxicological effects induced on early life stages of zebrafish (*Danio rerio*) after an acute exposure to microplastics alone or co-exposed with copper. Chemosphere.

[6] Paulino AT (2006). Novel adsorbent based on silkworm chrysalides for removal of heavy metals from wastewaters. J. Colloid Interface Sci..

[7] Ahuja AK, Singh P, Singh V (2019). Physico-chemical characterization of ground water with reference to water quality index and their seasonal variation in vicinity of thermal power plant at Yamuna Nagar, Haryana. Int. J. Adv. Sci. Res. Manage..

[8] Watari T (2021). Anaerobic biological treatment of EG/PG water-soluble copolymer coupled with down-flow hanging sponge reactor. Environ. Technol. Innov..

[9] Goodarzvand Chegini Z, Hassani H, Torabian A, Borghei SM (2020). Enhancement of PMS activation in an UV/ozone process for cyanide degradation: A comprehensive study. Pigment Resin Technol..

[10] Lapointe M, Farner JM, Hernandez LM, Tufenkji N (2020). Understanding and improving microplastic removal during water treatment: Impact of coagulation and flocculation. Environ. Sci. Technol..

[11] Liakos EV, Mone M, Lambropoulou DA, Bikiaris DN, Kyzas GZ (2021). Adsorption evaluation for the removal of nickel, mercury, and barium ions from single-component and mixtures of aqueous solutions by using an optimized biobased chitosan derivative. Polymers.

[12] Imron MF, Kurniawan SB, Titah HS (2019). Potential of bacteria isolated from diesel-contaminated seawater in diesel biodegradation. Environ. Technol. Innov..

[13] Syaichurrozi I, Sarto S, Sediawan WB, Hidayat M (2021). Effect of current and initial ph on electrocoagulation in treating the distillery spent wash with very high pollutant content. Water (Switzerland).

[14] Sikdar D, Goswami S, Das P (2020). Activated carbonaceous materials from tea waste and its removal capacity of indigo carmine present in solution: Synthesis, batch and optimization study. Sustain. Environ. Res..

[15] Narendrakumar G, Senthil P (2020). Adsorption of Chromium from aqueous solution by lignocellulosic biomass (*Pinus palustris*): Studies on equilibrium isotherm, and kinetics. J. Environ. Treat. Tech..

[16] Buema G, Maria H, Nicoleta L, Horia C, Loredana F, Gabriela C, Daniel B, Roxana DB (2021). Adsorption performance of modified fly ash for copper ion removal from aqueous solution. Water.

[17] Mehta D, Mondal P, George S (2016). Utilization of marble waste powder as a novel adsorbent for removal of fluoride ions from aqueous solution. J. Environ. Chem. Eng..

[18] Rahimian R, Zarinabadi S (2020). A review of studies on the removal of methylene blue dye from industrial wastewater using activated carbon adsorbents made from almond bark. Prog. Chem. Biochem. Res. J. Homepage.

[19] Qiu YW, Wang DX, Zhang G (2020). Assessment of persistent organic pollutants (POPs) in sediments of the Eastern Indian Ocean. Sci. Total Environ..

[20] Ren X, Guangming Z, Lin T, Jingjing W, Jia W, Yani L, Jiangfang Y, Huan Y, Shujing Y, Rui D (2018). Sorption, transport and biodegradation—An insight into bioavailability of persistent organic pollutants in soil. Sci. Total Environ..

[21] Ilhan F, Ulucan-Altuntas K, Avsar Y, Kurt U, Saral A (2019). Electrocoagulation process for the treatment of metal-plating wastewater: Kinetic modeling and energy consumption. Front. Environ. Sci. Eng..

[22] Pandiarajan A, Kamaraj R, Vasudevan S (2017). Enhanced removal of cephalosporin based antibiotics (CBA) from water by one-pot electrosynthesized Mg(OH)_2_: A combined theoretical and experimental study to pilot scale. New J. Chem..

[23] Aravind P, Selvaraj H, Ferro S, Sundaram M (2016). An integrated (electro- and bio-oxidation) approach for remediation of industrial wastewater containing azo-dyes: Understanding the degradation mechanism and toxicity assessment. J. Hazard. Mater..

[24] Moussa DT, El-Naas MH, Nasser M, Al-Marri MJ (2017). A comprehensive review of electrocoagulation for water treatment: Potentials and challenges. J. Environ. Manag..

[25] Myllymäki P, Lahti R, Romar H, Lassi U (2018). Removal of total organic carbon from peat solution by hybrid method—Electrocoagulation combined with adsorption. J. Water Process Eng..

[26] Panizza M, Cerisola G (2010). Applicability of electrochemical methods to carwash wastewaters for reuse. Part 2: Electrocoagulation and anodic oxidation integrated process. J. Electroanal. Chem..

[27] Inal IIG, Holmes SM, Banford A, Aktas Z (2015). The performance of supercapacitor electrodes developed from chemically activated carbon produced from waste tea. Appl. Surf. Sci..

[28] Gokce Y, Aktas Z (2014). Nitric acid modification of activated carbon produced from waste tea and adsorption of methylene blue and phenol. Appl. Surf. Sci..

[29] Mondal MK (2010). Removal of Pb(II) from aqueous solution by adsorption using activated tea waste. Korean J. Chem. Eng..

[30] Çelebi H, Gök G, Gök O (2020). Adsorption capability of brewed tea waste in waters containing toxic lead(II), cadmium (II), nickel (II), and zinc(II) heavy metal ions. Sci. Rep..

[31] Singh Thakur L, Parmar M, Parmar M (2013). Adsorption of heavy metal (Cu^2+^, Ni^2+^ and Zn^2+^) from synthetic waste water by tea waste adsorbent. Int. J. Chem. Phys. Sci..

[32] Elabbas S (2020). Eggshell adsorption process coupled with electrocoagulation for improvement of chromium removal from tanning wastewater. Int. J. Environ. Anal. Chem..

[33] Patil CS (2019). Waste tea residue as a low cost adsorbent for removal of hydralazine hydrochloride pharmaceutical pollutant from aqueous media: An environmental remediation. J. Clean. Prod..

[34] Jiang D (2019). Removal of the heavy metal ion nickel (II) via an adsorption method using flower globular magnesium hydroxide. J. Hazard. Mater..

[35] Khan J (2021). Removal of copper ions from wastewater via adsorption on modified hematite (α-Fe_2_O_3_) iron oxide coated sand. J. Clean. Prod..

[36] Jeppu GP, Clement TP (2012). A modified Langmuir–Freundlich isotherm model for simulating pH-dependent adsorption effects. J. Contam. Hydrol..

[37] Hussin F, Aroua MK, Szlachtac M (2019). Combined solar electrocoagulation and adsorption processes for Pb(II) removal from aqueous solution. Chem. Eng. Process. Process Intensif..

[38] Nigri EM, Santos ALA, Rocha SDF (2020). Removal of organic compounds, calcium and strontium from petroleum industry effluent by simultaneous electrocoagulation and adsorption. J. Water Process Eng..

[39] GilPavas E, Correa-Sanchez S (2020). Assessment of the optimized treatment of indigo-polluted industrial textile wastewater by a sequential electrocoagulation-activated carbon adsorption process. J. Water Process Eng..

[40] Yıldız S, Çekim M, Dere T (2017). Biosorption of Cu^2+^ and Ni^2+^ Ions from synthetic waters. Appl. Biochem. Biotechnol..

[41] Nikolic M, Jeffry Robert R, Girish CR (2019). The adsorption of cadmium, nickel, zinc, copper and lead from wastewater using tea fiber waste. J. Eng. Appl. Sci..

[42] Nigam M, Rajoriya S, Rani Singh S, Kumar P (2019). Adsorption of Cr (VI) ion from tannery wastewater on tea waste: Kinetics, equilibrium and thermodynamics studies. J. Environ. Chem. Eng..

[43] Ahmaruzzaman M, Gayatri SL (2010). Activated tea waste as a potential low-cost adsorbent for the removal of p-nitrophenol from wastewater. J. Chem. Eng. Data.

[44] Cherdchoo W, Nithettham S, Charoenpanich J (2019). Removal of Cr(VI) from synthetic wastewater by adsorption onto coffee ground and mixed waste tea. Chemosphere.

[45] Uzun BB, Apaydin-Varol E, Ateş F, Özbay N, Pütün AE (2010). Synthetic fuel production from tea waste: Characterisation of bio-oil and bio-char. Fuel.

[46] Nemeş L, Bulgariu L (2016). Optimization of process parameters for heavy metals biosorption onto mustard waste biomass. Open Chem..

[47] Boujelben N, Bouzid J, Elouear Z (2009). Adsorption of nickel and copper onto natural iron oxide-coated sand from aqueous solutions: Study in single and binary systems. J. Hazard. Mater..

[48] Al-Qodah Z, Al-Shannag M (2017). Heavy metal ions removal from wastewater using electrocoagulation processes: A comprehensive review. Sep. Sci. Technol. (Philadelphia).

[49] Ramesh TN, Kirana DV, Ashwini A, Manasa TR (2017). Calcium hydroxide as low cost adsorbent for the effective removal of indigo carmine dye in water. J. Saudi Chem. Soc..

[50] Can BZ, Boncukcuoglu R, Yilmaz AE, Fil BA (2014). Effect of some operational parameters on the Arsenic removal by electrocoagulation using iron electrodes. J. Environ. Health Sci. Eng..

[51] Bazrafshan E, Mohammadi L, Ansari-Moghaddam A, Mahvi AH (2015). Heavy metals removal from aqueous environments by electrocoagulation process—A systematic review. J. Environ. Health Sci. Eng..

[52] Ibrahim AG, Saleh AS, Elsharma EM, Metwally E, Siyam T (2019). Chitosan-g-maleic acid for effective removal of copper and nickel ions from their solutions. Int. J. Biol. Macromol..

[53] Liu S (2017). Facile synthesis of Cu(II) impregnated biochar with enhanced adsorption activity for the removal of doxycycline hydrochloride from water. Sci. Total Environ..

[54] Akansha J, Nidheesh PV, Gopinath A, Anupama KV, Suresh Kumar M (2020). Treatment of dairy industry wastewater by combined aerated electrocoagulation and phytoremediation process. Chemosphere.

[55] Abbaszadeh S, Wan Alwi SR, Webb C, Ghasemi N, Muhamad II (2016). Treatment of lead-contaminated water using activated carbon adsorbent from locally available papaya peel biowaste. J. Clean. Prod..

[56] Pizutti JT, de Dos Santos RC, Hemkemeier M, Piccin JS (2019). Electrocoagulation coupled adsorption for anaerobic wastewater post-treatment and reuse purposes. Desalin. Water Treat..

[57] Zakaria MR, Abdul Kudus MH, Md. Akil H, Mohd Thirmizir MZ (2017). Comparative study of graphene nanoparticle and multiwall carbon nanotube filled epoxy nanocomposites based on mechanical, thermal and dielectric properties. Compos. Part B Eng..

[58] Ziouvelou A, Tekerlekopoulou AG, Vayenas DV (2019). A hybrid system for groundwater denitrification using electrocoagulation and adsorption. J. Environ. Manag..

[59] Barhoumi A (2019). High-rate humic acid removal from cellulose and paper industry wastewater by combining electrocoagulation process with adsorption onto granular activated carbon. Ind. Crops Prod..

[60] Vieno N, Tuhkanen T, Kronberg L (2006). Removal of pharmaceuticals in drinking water treatment: Effect of chemical coagulation. Environ. Technol..

[61] Al-Qodah Z, Yahya MA, Al-Shannag M (2017). On the performance of bioadsorption processes for heavy metal ions removal by low-cost agricultural and natural by-products bioadsorbent: A review. Desalin. Water Treat..

[62] Jiang Y, Pang H, Liao B (2009). Removal of copper(II) ions from aqueous solution by modified bagasse. J. Hazard. Mater..

[63] Reck IM, Paixão RM, Bergamasco R, Vieira MF, Vieira AMS (2018). Removal of tartrazine from aqueous solutions using adsorbents based on activated carbon and *Moringa oleifera* seeds. J. Clean. Prod..

[64] Khosla E, Kaur S, Dave PN (2013). Tea waste as adsorbent for ionic dyes. Desalin. Water Treat..

[65] Wagle D, Lin CJ, Nawaz T, Shipley HJ (2020). Evaluation and optimization of electrocoagulation for treating Kraft paper mill wastewater. J. Environ. Chem. Eng..

[66] Hendaoui K (2018). Real indigo dyeing effluent decontamination using continuous electrocoagulation cell: Study and optimization using Response Surface Methodology. Process Saf. Environ. Prot..

[67] Current Wastewater Sludge Treatment Situation in Shanghai, Beijing, Chongqing (2011).

[68] Li LY, Gong XD, Abida O (2019). Waste-to-resources: Exploratory surface modification of sludge-based activated carbon by nitric acid for heavy metal adsorption. Waste Manag..

[69] Wan S (2014). Sorption of lead(II), cadmium(II), and copper(II) ions from aqueous solutions using tea waste. Ind. Eng. Chem. Res..

[70] Nejadshafiee V, Islami MR (2019). Adsorption capacity of heavy metal ions using sultone-modified magnetic activated carbon as a bio-adsorbent. Mater. Sci. Eng. C.

[71] Tran, T. Van, Thi, Q. & Bui, P. A comparative study on the removal efficiency of metal using sugarcane bagasse—derived ZnCl_2_—activated carbon by the response surface methodology. (2017). 10.1177/0263617416669152.

[72] Shrestha RM, Joshi S (2019). Application of coconut shell for the preparation of activated carbon to remove heavy metal from aqueous solution. J. Adv. Eng..

[73] Landin-sandoval VJ, Mendoza-castillo DI, Bonilla-petriciolet A, Aguayo-villarreal IA (2020). Valorization of agri-food industry wastes to prepare adsorbents for heavy metal removal from water. J. Environ. Chem. Eng..

[74] Alsadi J (2019). Synthesis of activated carbon from sugarcane bagasse and application for mercury adsorption. Pollution.

